# Evaluation of clinical research priorities in Asian intensive care units (ERA-ICU)

**DOI:** 10.1186/s40560-025-00816-9

**Published:** 2025-08-27

**Authors:** Andrew Li, Prashant Nasa, Sheila Nainan Myatra, Gentle Sunder Shrestha, Abdulrahman Al-Fares, Ming-Cheng Chan, Young-Jae Cho, Moritoki Egi, Mohammad Omar Faruq, Carine Harmouche, Seyed Mohammad Reza Hashemian, Ayman Kharaba, Aidos Konkayev, Faisal Muchtar, Khalid Mahmood Khan Nafees, Mendsaikhan Naranpurev, Do Ngoc Son, Pauline Yeung Ng, Mohd Basri Mat-Nor, Jose Emmanuel Palo, Mehmet Uyar, Zhongheng Zhang, Jigeeshu Divatia, Jason Phua, Yaseen M. Arabi, Lowell Ling, the ERA-ICU Study Group, the Asian Critical Care Clinical Trials Group, Andrew Li, Prashant Nasa, Sheila Nainan Myatra, Gentle Sunder Shrestha, Abdulrahman Al-Fares, Ming-Cheng Chan, Young-Jae Cho, Moritoki Egi, Mohammad Omar Faruq, Carine Harmouche, Seyed Mohammad Reza Hashemian, Ayman Kharaba, Aidos Konkayev, Faisal Muchtar, Khalid Mahmood Khan Nafees, Mendsaikhan Naranpurev, Pauline Yeung Ng, Mohd Basri Mat-Nor, Jose Emmanuel Palo, Mehmet Uyar, Zhongheng Zhang, Jigeeshu Divatia, Jason Phua, Yaseen M. Arabi, Lowell Ling, Ariful Basher, Mahbub Morshed, Tasmia Kashfi, Bin Lin, Chun Pan, Dechang Chen, Gang Wang, Jiao Liu, Lihui Wang, Ling-Ai Pan, Lining Si, Xu Wei, Yi Zhang, Yiping Wang, Yuetian Yu, Hoi Ping Shum, Kwok-Ming Ho, Man Yee Man, Wai-Tat Wong, Anirban Hom Choudhuri, Badri Prasad Das, Bharat G. Jagiasi, Deepak Govil, Mehul Shah, Pravin Amin, Rajesh Mishra, Rajesh Mohan Shetty, Ravi Shankar, Shivangi Mishra, Simant Jha, Subhal Dixit, Swarna Deepak Kuragayala, R. Vaidyanathan, Ziyokov Joshi, Erwin Pradian, Haizah Nurdin, Nurita Dian Kestriani Saragi Sitio, Hideto Yasuda, Kazuaki Atagi, Benazir Azimova, Maiya Konkayeva, Nursultan Dauletbaev, Dalal Aldosari, Sajida Sange, Zeina Aoun Bacha, Ji Zhang Chin, M Shanaz Hasan, Muhamad Hafizzi, Nor’azim Mohd Yunos, Zheng-Yii Lee, Enkhsaikhan Samdan, Erdenechimeg Tegshee, Suvd-Erdene Narmandakh, Tamir Lkhagvadorj, Telmen Amartur, Tsolmon Begzjav, Mahir AlBahrani, Ashok Kumar, Muhammad Sohaib, Muneeb Ali, Sheharyar Ashraf, Debbie Noblezada-Uy, Faith Joan Gaerlan, Gerardo Briones, Jeremiah Butch T. Gemarino, Kevin De Asis, Marion Patricio, Pauline Convocar, Rodolfo Roman Bigornia, Byunghyuk Yu, Jae Kyeom Sim, Jongmin Lee, Jung-Min Bae, Kyoung Hoon Lim, Kyungsoo Chung, Soyoung Park, Sejoong Kim, Song I. Lee, Won-Young Kim, Abdullah M. Alhammad, Carlos Sanchez, Faten Farid Awdallah, Ghaleb Almekhlafi, Mohamed Hegazy, Mohammed Alshahrani, Rawah Shafiq Aljishi, Samiyah Alanazi, Zainab Al Duhailib, Amartya Mukhopadhyay, Balachandran Kayachandran, Charles Chin Han Lew, Clarabella Liew, Geetha Kayambu, John Tsoong, K. Ramanathan, Kay Choong See, Kumaresh Venkatesan, Matthew Cove, Roshni Gokhale, Sharlene Ho, Shir Lynn Lim, Yew Woon Chia, Yi Hern Tan, Yie Hui Lau, Yu-Lin Wong, Will Loh, Cong-Tat Cia, Han-Chung Hu, Jia-Jun Wu, Jia-Yih Feng, Ming-Chieh Yang, Sheng-Yuan Ruan, Alaaeiden Ghanem, Imadeddin Barakat, Jihad Mallat, Rania Omar, Reda Mohamed Sherif, Binh Son Ha, Chinh Quoc Luong, Dai Quang Huynh, Hieu Huu Hoang, Huan Huu Nguyen, Hung Ngoc Dinh, Hung Tan Nguyen, Minh Hoa Le, Kien Trung Nguyen, Phuoc Thien Duong, Son Ngoc Do, Dung Tat Nguyen, Thai Van Hoang, Thao Thi Ngoc Pham, Thien Xuan Mai, Thuy Thi Phuong Le, To Dang Nguyen, Chinh Huy Vu

**Affiliations:** 1 Department of Respiratory and Critical Care Medicine, Tan Tock Seng Hospital, Singapore, Singapore; 2 Department of Intensive Care Medicine, Woodlands Health, Singapore, Singapore; 3 Critical Care Medicine, NMC Specialty Hospital, Dubai, United Arab Emirates; 4 Integrated Critical Care Unit, New Cross Hospital, The Royal Wolverhampton NHS Trust, Wolverhampton, UK; 5 Department of Anaesthesiology, Critical Care and Pain, Tata Memorial Hospital, Homi Bhabha National Institute, Mumbai, Maharashtra India; 6 Department of Critical Care Medicine, Institute of Medicine, Tribhuvan University Teaching Hospital, Kathmandu, Bagmati Nepal; 7 Australian and New Zealand Intensive Care Research Centre (ANZIC-RC), School of Public Health and Preventive Medicine, Monash University, Melbourne, VIC Australia; 8 Department of Anesthesia, Critical Care Medicine, and Pain Medicine, Ministry of Health, Al-Amiri Hospital, Kuwait City, Kuwait; 9 Kuwait Extracorporeal Life Support Program, Al-Amiri Center for Respiratory and Cardiac Failure, Ministry of Health, Kuwait City, Kuwait; 10 Department of Critical Care Medicine, Taichung Veterans General Hospital, Taichung, Taiwan; 11 Department of Post-Baccalaureate Medicine, College of Medicine, National Chung Hsing University, Taichung, Taiwan; 12 Division of Critical Care and Respiratory Therapy, Department of Internal Medicine, Taichung Veterans General Hospital, Taichung, Taiwan; 13 Division of Pulmonary and Critical Care Medicine, Department of Internal Medicine, Seoul National University Bundang Hospital, Seoul National University College of Medicine, Seongnam, Republic of Korea; 14 Department of Anesthesia, Kyoto University Hospital, Kyoto, Japan; 15 Department of Critical Care Medicine & Emergency Medicine, United Hospital, Dhaka, Bangladesh; 16 Pulmonary & Critical Care Division, Hotel Dieu de France, Saint Joseph University of Beirut, Beirut, Lebanon; 17 Chronic Respiratory Disease Research Center, National Research Institute of Tuberculosis and Lung Diseases, Masih Daneshvari Hospital, Shahid Beheshti University of Medical Sciences, Tehran, Iran; 18 Department of Critical Care, King Fahad Hospital, Al Madinah Al Monawarah, Saudi Arabia; 19 Anaesthesiology and Intensive Care Department, Astana Medical University, Astana, Kazakhstan; 20 National Science Center of Traumatology and Orthopedia Named Batpenov, Astana, Kazakhstan; 21 Department of Anesthesiology, Intensive Care and Pain Management, Medical Faculty, Hasanuddin University, Makassar, South Sulawesi Indonesia; 22 Ministry of Health, Department of Critical Care Medicine, RIPAS Hospital, Bandar Seri Begawan, Brunei; 23 Intensive Care Department, Mongolia Japan Hospital, Mongolian National University of Medical Sciences, Ulaanbaatar, Mongolia; 24 Center for Critical Care Medicine, Bach Mai Hospital, Hanoi, Vietnam; 25 Department of Emergency and Critical Care Medicine, Faculty of Medicine, VNU University of Medicine and Pharmacy, Vietnam National University, Hanoi, Vietnam; 26 Department of Emergency and Critical Care Medicine, Hanoi Medical University, Hanoi, Vietnam; 27 Critical Care Medicine Unit, The University of Hong Kong, Hong Kong SAR, China; 28 Department of Adult Intensive Care, Queen Mary Hospital, Hong Kong SAR, China; 29 Kulliyyah of Medicine, International Islamic University Malaysia, Kuantan, Pahang Malaysia; 30 Acute and Critical Care Institute, The Medical City, Pasig, Philippines; 31 Department of Anaesthesiology and Intensive Care Unit, Ege University School of Medicine Hospital, Izmir, Turkey; 32 Department of Emergency Medicine, Provincial Key Laboratory of Precise Diagnosis and Treatment of Abdominal Infection, Sir Run Run Shaw Hospital, Zhejiang University School of Medicine, Hangzhou, China; 33 Critical Care Medicine, Lilavati Hospital and Research Centre, Mumbai, India; 34 Division of Respiratory and Critical Care Medicine, Department of Medicine, Alexandra Hospital, National University Health System, Singapore, Singapore; 35 Division of Respiratory and Critical Care Medicine, National University Hospital, National University Health System, Singapore, Singapore; 36 Department of Medicine, Yong Loo Lin School of Medicine, National University of Singapore, Singapore, Singapore; 37 College of Medicine, King Abdullah International Medical Research Center, Intensive Care Department, King Abdulaziz Medical City, Ministry of National Guard Health Affairs, King Saud Bin Abdulaziz University for Health Sciences, Riyadh, Saudi Arabia; 38 Department of Anaesthesia and Intensive Care, The Chinese University of Hong Kong, Hong Kong SAR, China; 39 Department of Intensive Care, Prince of Wales Hospital, Hong Kong SAR, China

**Keywords:** Critical care, Investigation, Agenda, Interest, Feasibility, Importance, International, Clinical study, Relevance

## Abstract

**Background:**

Practice and delivery of critical care in Asia varies according to healthcare structure, income setting, and cultural factors. Identifying research priorities specific to ICU patients and healthcare workers in Asia is needed to guide advancement of critical care in the region.

**Methods:**

This was an international cross-sectional survey study with adapted methods from nominal group techniques. All members of the Asian Critical Care Clinical Trials (ACCCT) Group were invited to submit research question suggestions. Submitted research questions were combined into summarized research questions, grouped into research themes, and individually ranked by number of mentions based on the original question submission (popularity). National and Regional Representatives rated the top 15% most popular summarized research questions by pre-defined importance and feasibility criteria.

**Results:**

Between September 20, 2024 and December 10, 2024, 160 of 228 general members of the ACCCT Group (response rate 70.2%) participated in this survey study. The participants were from 112 hospitals across 24 countries and regions within Asia. Participants submitted 408 research questions, which were categorized into 15 themes and combined into 197 summarized research questions. The top three themes, as ranked by the number of mentions, were infection/sepsis, general ICU care, and structure/training/staffing/teamwork/safety. A threshold of 4 mentions was used to identify 26 summarized research questions that represented the top 15% most popular questions. Research questions related to sepsis and acute respiratory distress syndrome were ranked most important and feasible across the region.

**Conclusion:**

Twenty-six of the most popular research questions in critical care were identified by Asian ICU workers and researchers to drive research agenda in Asia for the next decade.

**Supplementary Information:**

The online version contains supplementary material available at 10.1186/s40560-025-00816-9.

## Introduction

The Asian Critical Care Clinical Trials (ACCCT) Group is a research collaboration of intensive care unit (ICU) clinicians and researchers from Asia [1]. The mission of the group is to “improve the practice of critical care medicine and the outcomes of critically ill patients through collaborative clinical research in Asia.” Since its formation in 2012, the group has conducted the MOSAICS I, MOSAICS II, ACME, SABA, AISPO, Asian ABC, Asian ABC2, and IMPROVE studies [2–8]. The research areas have ranged from sepsis epidemiology to evaluation of end-of-life care practices in Asian ICUs.

Compared to other national critical care trial groups and networks, the group comprises representation from 28 individual countries and regions that span all income groups. Although the diversity within the ACCCT Group is a key strength, it also presents unique challenges in clinical research, including variability in research capacity and a lack of centralized funding. In addition, differences in case-mix, healthcare structure as well as geographical and cultural factors may affect research priorities from individual countries and regions. Thus far, the studies conducted by the ACCCT Group have been exclusively investigator-led and initiated by individual researcher interests [2–6].

The ACCCT Group’s vision is to “inspire research question on clinically important problems faced by Asian intensive care units.” Internationally, research priority studies have been conducted to foster collaborative efforts and drive investigative agendas in critical care, encompassing multiple areas such as prehospital critical care, pediatric intensive care, critical care nursing, acute respiratory distress syndrome (ARDS) and sepsis [9–13]. However, these studies have been exclusively focused on research priorities in western high-income countries. While some of the identified clinical problems are universal and likely encountered in Asian ICUs as well, research priorities specific to ICU patients and healthcare workers in the Asian healthcare setting are absent. The objective of the Evaluation of Importance and Feasibility of clinical research questions in Asian Intensive Care Units (ERA-ICU) study is to identify a set of research topics that are ranked by priority and feasibility to inform the research agenda for Asian ICUs over the next 5–10 years.

## Methods

### Study design

This was an international cross-sectional survey study with adapted methods from nominal group techniques [14]. All members of the ACCCT Group were invited to take part in this study to drive the research agenda for the group and the wider Asian critical care community. The study was conducted over three phases. In the first phase, the study steering committee communicated via electronic mail and met online to define the importance and feasibility criteria that were used to prioritize research questions in subsequent phases. In the second phase, all ACCCT Group general members were invited to suggest research questions. In the third phase, National/Regional representatives ranked the most popular research questions to prioritize the research agenda according to the pre-determined importance and feasibility criteria. The conduct and reporting of this cohort study followed the Reporting Guideline for Research Priority Setting (REPRISE) [15]. The study was approved by the Survey and Behavioural Research Ethics Committee of the Chinese University of Hong Kong (SBRE-23-0325).

### Study steering committee

A study steering committee was convened to design and conduct the study. Membership included the ACCCT Group Executive Committee and experienced ICU researchers within the ACCCT Group who have individually led multi-center clinical studies and coordinated research projects. The steering group was responsible for defining importance and feasibility criteria, designing the survey, summarizing and refining research topics, categorizing research themes, analyzing the data, and reporting the study findings.

### National/Regional representatives

National/Regional representatives are general members of the ACCCT Group who self-nominated and were selected by the Executive Committee of the ACCCT Group to champion the needs and research priorities of individual countries and regions. Selection was based on track record in clinical research and diversity in terms of age and gender. They were responsible for ensuring participation from their respective country/region and ranking the summarized research questions based on importance and feasibility criteria.

### General members of the ACCCT group

ACCCT group general members are healthcare workers or researchers working in ICUs in Asia. All self-registered general members were invited to participate in the study.

### Scope of research questions

The scope of the study was to develop a set of research questions that address the knowledge gap in the clinical diagnosis and management of critically ill patients, as well as the epidemiology and pathophysiology of critical illness syndromes, staff and healthcare organizational structures, within the context of Asian ICUs. Research topics should have a particular emphasis on topics important to, but not necessarily unique to Asian ICUs. The intended beneficiaries are critically ill patients and ICU healthcare staff in Asia. The goal is to identify relevant research priorities that can be addressed within the next decade.

### Phase 1: setting importance and feasibility criteria

Each member of the study steering committee was asked to generate a preliminary list of components that should be included in the importance criteria and feasibility criteria. The steering committee then met online to discuss and finalize the components for each criterion (Table 1). The purpose of the importance criteria was to inform the relative importance ranking of summarized research questions in the third round. The feasibility criteria provided guidance on assessing the practicality of conducting research studies to address the summarized research questions within the Asian ICU setting.

**Table 1:** Importance and feasibility criteria

Importance criterion	Explanation
Novelty	The research question/study has not already been addressed fully by existing studies
Uniqueness of Asian critical care	The research question/study is especially relevant for Asia due to the unique biology of Asian patients and the unique circumstances of Asian critical care
Disease and/or process burden	The research question/study focuses on diseases and/or processes that have a high burden in Asia as far as number of patients, costs of care, and outcomes are concerned
Anchor programme	The research question/study is part of a larger research programme that shows coherence and a long-term view
Future impact	The research question/study allows translation of findings into policy and practice that will benefit patients and healthcare workers in critical care
Generalizability and relevance across income settings	The research question/study produces findings that can be generalized to multiple income settings
Inclusiveness/collaboration	The research question/study enhances real and long-term collaboration between many countries/regions, ICUs, researchers, and healthcare workers
Upleveling	The research question/study facilitates the upleveling and upskilling of researchers, healthcare workers, and ICUs in research, clinical, and administrative matters

Feasibility criterion	Explanation
Financial costs	The research question/study does not require external funding or has already secured financial support
Resources	The research question/study uses locally available resources instead of requiring additional support that would make both the study hard to conduct and the findings hard to implement
Ethics approval	The research question/study will be cleared by local, regional, and national ethics boards
Recruitment	The research question/study encourages seamless recruitment of subjects and ICUs
Inter-country/region differences	The research question/study can be conducted across many countries/regions despite differences in their research climate, patient mix, ICU practices, and healthcare systems
Timeframe	The research question/study is feasibly addressed within 3 years
Cultural awareness	The research question/study should be sensitive to differences in religion and cultural issues in Asia
Expertise	The trial group has the appropriate skillset and expertise to complete the study

Components of the importance and feasibility criteria were drafted by the study steering committee. National/regional representatives used these criteria to rate the importance and feasibility of the most popular research questions in phase 3 of the study

### Phase 2: generate research questions

All general members and National/Regional Representatives of the ACCCT Group were invited to participate in an online cross-sectional survey to suggest research questions (Appendix 1). Guidance was provided to construct research questions according to the scope of this study (Appendix 2). The survey was administered using REDCap (version 14.3.11) with weekly reminders. Thereafter, the study steering committee grouped the suggested research questions into broad themes. The committee also combined and refined the suggested research questions that were similar and related into a set of summarized questions. Each summarized question was assigned a popularity score based on the number of times it was mentioned from the list of research questions submitted by the ACCCT Group general members at the beginning of Phase 2. The questions were ordered by popularity, and the top 15% of research questions were brought forward to Phase 3.

### Phase 3: ranking research questions based on importance and feasibility

Each ACCCT Group National/Regional Representative voted on all components of the importance and feasibility criteria for the top 15% of summarized questions identified from Phase 2 using a 7-point Likert scale: 7—strongly agree, 6—agree, 5—somewhat agree, 4—neutral, 3—somewhat disagree, 2—disagree, and 1—strongly disagree. Mean scores were used to summarize votes for the importance and feasibility criteria. The overall importance and feasibility score was calculated by averaging all components of the importance criteria and feasibility criteria and then multiplying by 100, respectively. The priority ranking of summary research questions was based on the overall importance and feasibility scores (higher score equated to higher priority). During this phase, the popularity score for each summarized question was revealed to the National/Regional Representatives.

### Statistics

Response rate was calculated by dividing the number of respondents by the total number of general members in the ACCCT Group. Descriptive statistics such as median and interquartile range and percentages were used to describe the demographics of study participants. Popularity of summarized questions was calculated by counting the number of times the summarized question was mentioned among all submitted research questions. Frequency of number of mentions for all summary research questions was calculated. The most popular research questions were identified by selecting a threshold for number of mentions that approximated to the top 15% of questions as ordered by number of mentions. Themes were ranked by total number of mentions from included questions. Phase 2 and 3 were administered using REDCap (version 14.3.11) with weekly reminders. All statistical analyses and plots were performed and drawn in R Studio (version 2024.04.2 + 764).

## Results

### Study participants

**Fig. 1:**
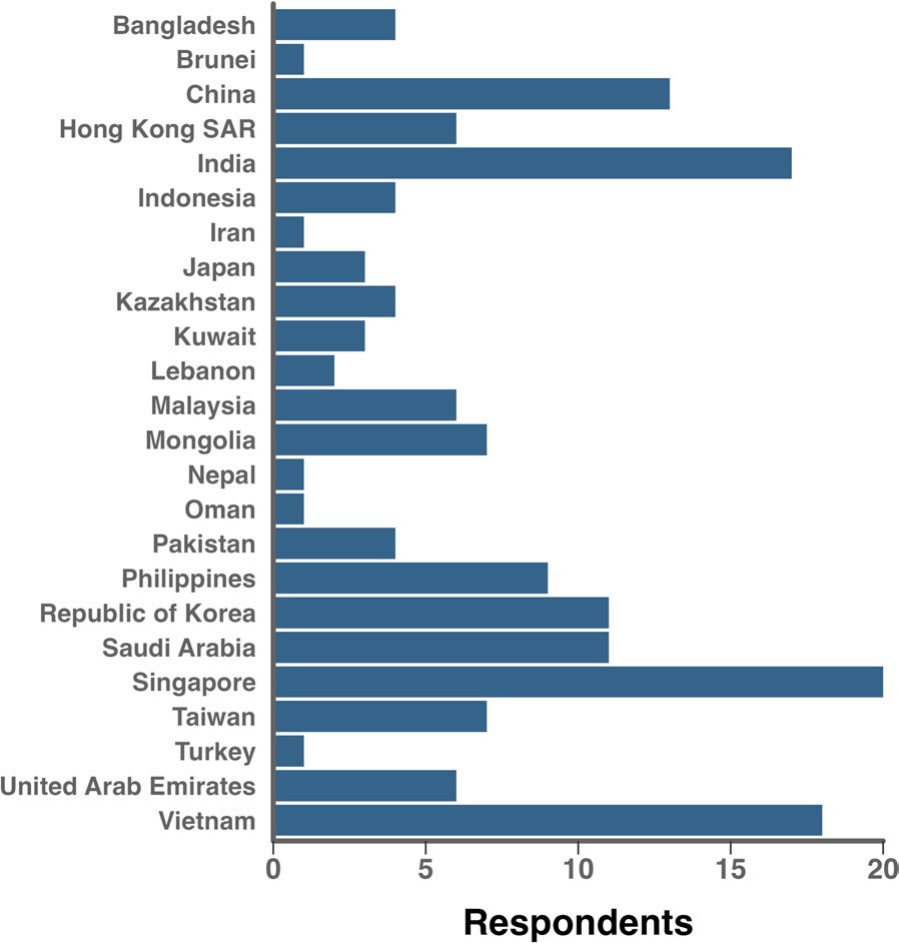
Geographical distribution of participants. The geographical distribution of phase 2 survey participants

Between September 20, 2024, and December 10, 2024, 160 of 228 general members of the ACCCT Group participated in Phase 1 of this study (response rate 70.2%) (Appendix 3). The participants were from 112 hospitals across 24 countries and regions within Asia (Fig. 1). The demographics of study participants are shown in Table 2. The median age was 46 (40–50) years old, and 39% of participants were female. The median duration of ICU experience was 15 (10–20) years. Most of the participants were physicians (95.0%). Participants indicated that they were interested in conducting survey (82%), observational (88%) and interventional (70%) studies.

**Table 2:** Participant demographics

	*n* = 160
Age (years)	46 (40 – 50)
Female sex (%)	39 (24.4)
ICU experience (years)	15 (10–20)
Healthcare professional (%)	
Dietician	2 (1.3)
Doctor	152 (95.0)
Nurse	2 (1.3)
Pharmacist	2 (1.3)
Physiotherapist	2 (1.3)
Urban location (%)	153 (95.6)
Type of hospital (%)	
General	39 (24.4)
Regional	12 (7.5)
Tertiary	109 (68.1)
Teaching affiliated (%)	134 (83.1)
Type of ICU (%)	
Cardiology/cardiothoracic	6 (4)
Medical	36 (23)
Mixed	105 (66)
Neurology/neurosurgical	1 (1)
Surgical	12 (8)
Interested study type (%)	
Survey	128 (80)
Observational	141 (88.1)
Interventional	114 (71.3)

All numbers are shown as median and interquartile range unless specified. Demographics of survey participants in Phase 2. *ICU* intensive care

### Submitted research questions

**Fig. 2:**
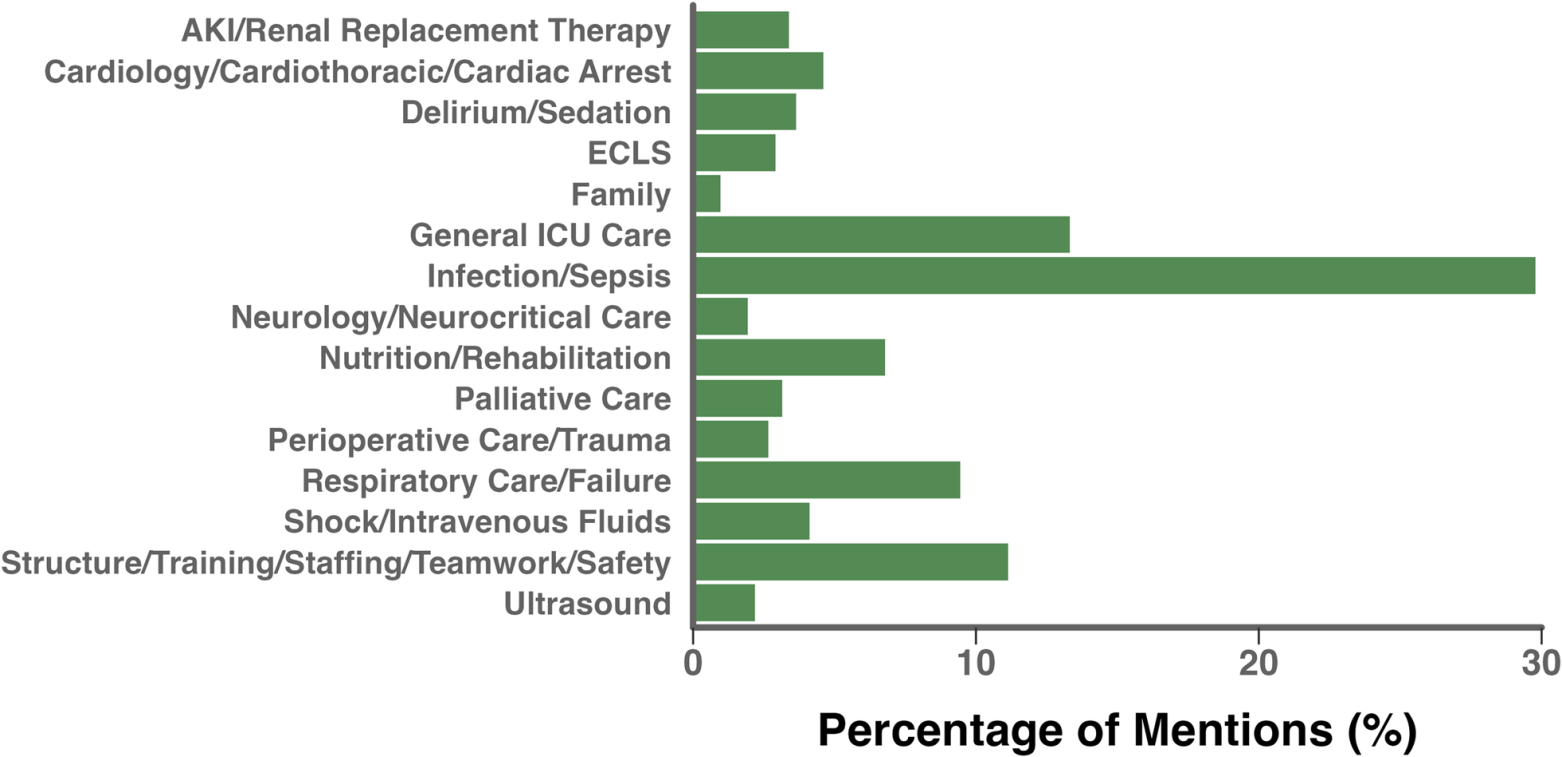
Popularity of research themes. The proportion of mentions from all suggested research questions accumulated by all research themes. *AKI* acute kidney injury, *ECLS* extracorporeal life support

Participants submitted 451 responses for research question suggestions in Phase 2. After excluding 43 responses that were not questions, 408 research questions were categorized into 15 themes: acute kidney injury/renal replacement therapy, cardiology/cardiothoracic/cardiac arrest, delirium/sedation, extracorporeal life support (ECLS), family, general ICU care, infection/sepsis, neurology/neurocritical care, nutrition/rehabilitation, palliative care, perioperative care/trauma, respiratory care/failure, shock/intravenous fluids, structure/training/staffing/teamwork/safety, and ultrasound. The steering committee combined and refined the submitted research questions, resulting in 197 summarized questions (Supplementary Table 1). These summarized questions were collectively mentioned 413 times by the original research question suggestions (some of the latter were related to more than one summarized question). The top three themes, based on the number of mentions, were infection/sepsis, general ICU care, and structure/training/staffing/teamwork/safety (Fig. 2 and Supplementary Table 2).

**Fig. 3:**
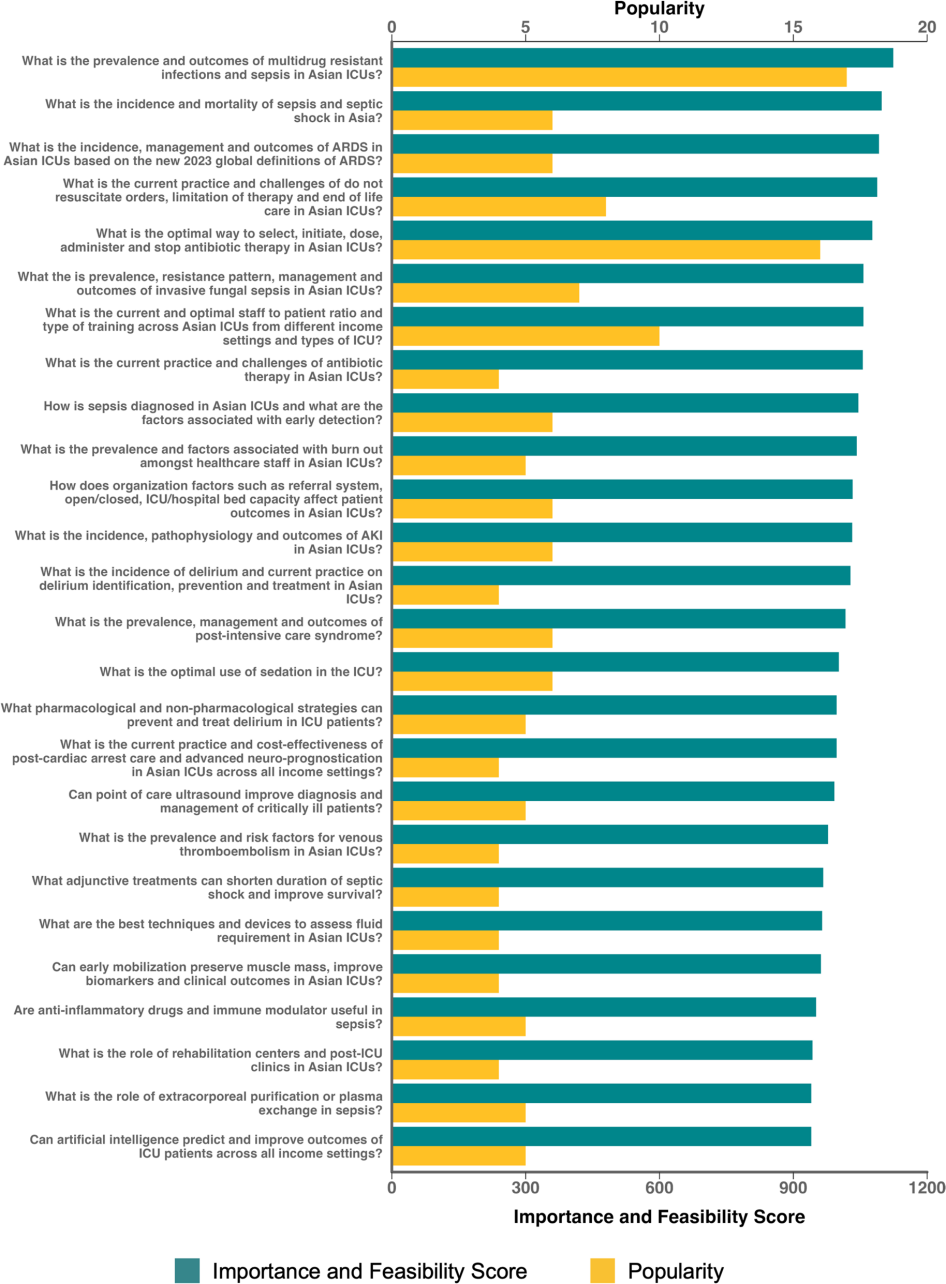
Priority ranking of top 26 summarized research questions. Priority ranking of the top 26 summarized research questions by overall importance and feasibility score. Popularity of each summarized research question is calculated from the number of mentions for each question from 408 original submitted research questions in phase 2

A threshold of 4 mentions was used to identify 26 research questions that were ranked in the top 15% of summarized research questions ordered by popularity (Fig. 3, Supplementary Table 3 and Supplementary Table 4). The 2 most popular questions were from the infection/sepsis theme: “What is the prevalence and outcomes of multidrug resistant infections and sepsis in Asian ICUs?” and “What is the optimal way to select, initiate, dose, administer and stop antibiotic therapy in Asian ICUs?” which accumulated 17 and 16 mentions, respectively (Supplementary Table 5). Themes that did not include at least one of the top 15% of research questions ranked by popularity included: ECLS, family neurology/neurocritical care, and perioperative care/trauma.

### Priority ranking of summarized research questions

All 21 ACCCT Group national/regional representatives (100% response rate) rated the importance and feasibility of the 26 most popular research questions from Phase 2 according to the pre-determined criteria (Fig. 3 and Supplementary Table 5). Mean scores for each component of the importance and feasibility criteria are shown in Supplementary Table 6 and Supplementary Table 7. Overall, the three research questions with the highest priority rank by summation of importance and feasibility scores were “What is the prevalence and outcomes of multidrug resistant infections and sepsis in Asian ICUs?”, “What is the incidence and mortality of sepsis and septic shock in Asia?” and “What is the incidence, management and outcomes of ARDS in Asian ICUs based on the new 2023 global definition of ARDS?”. The distributions of Likert ratings for importance and feasibility criteria of individual research questions are shown in Supplementary Fig. 1. Overall, the Likert distributions show that higher ranked research questions were more skewed towards agreement rather than disagreement for most components of both importance and feasibility compared to lower ranked questions.

## Discussion

In this international cross-sectional survey study involving 162 ICU healthcare workers from 112 hospitals across 24 countries and regions within Asia, we identified 26 summarized research questions that ranked in the top 15% of suggested research questions by popularity. The top three themes by popularity were infection/sepsis, general ICU care, and structure/training/staffing/teamwork/safety. The research questions with the highest priority were related to sepsis and ARDS, as ranked by pre-determined importance and feasibility criteria.

Our study showed that ICU workers in Asian ICUs were most interested in research questions related to sepsis, general ICU care, and organizational issues. This is somewhat surprising since sepsis and organizational issues have been the focus of many previous ACCCT Group studies [2, 3, 5–7, 16, 17]. Their current popularity not only validates the importance of these previous studies, but suggests these topics remain relevant and challenging in Asian ICUs. The MOSAICS II study has previously shown that 1 in 5 admissions to Asian ICUs are due to sepsis [2]. This may explain why infection- and sepsis-related research questions ranked as the most popular in our region. Organizational issues, such as optimal staffing ratio and training requirements, were also popular. This reflects the desire to optimize patient outcomes given the resource constraints in low-resourced settings and variations in organizational structure, staffing, and delivery of ICU care in our region [17]. Moreover, the Asian ABC study has revealed that ICU bed capacity may be relatively limited even in high-income countries/regions such as Japan and Hong Kong [5]. In fact, variable ICU bed capacity is also found across Europe [18]. Moreover, ways to optimize the ICU workforce have also been identified as a key research priority by the ICU community in the UK [18, 19].

The General ICU Care theme encompassed 32 suggested research questions, but many of these questions only had a few mentions. Therefore, although General ICU Care was a popular theme, only two questions were ranked among the top 15% most popular questions. The popularity of “Can artificial intelligence predict and improve outcomes of ICU patients across all income settings?” reflects artificial intelligence’s emerging position as a hot topic in medical research. But interestingly, this question ranked lowest for overall priority, primarily because it received the lowest score in terms of feasibility. This supports the opinion that AI’s potential to transform critical care is undeniable, but its implementation and practical utilization remain very limited [20].

In contrast, the infection/sepsis theme contained the highest number of summarized research questions which collectively accounted for 30% of the total number of mentions. The question “What is the prevalence and outcomes of multidrug resistant infections and sepsis in Asian ICUs?” was ranked most important and feasible. This was not particularly surprising, as an unequal burden of antimicrobial resistance (AMR) is predicted to inflict the South Asia region in the next few decades, particularly in low- to middle-income settings [21]. In addition, the recent EUROBACT II study showed that adequate antibiotics were provided in just over 50% of patients with bacteraemia even when 85% of the cohort was from upper-middle to high-income settings [22]. The problem is likely worse in Asia where there is a significant burden of AMR in low income settings coupled with limited access to expensive broad-spectrum antibiotics. There is a need to understand the barriers to providing the most basic sepsis care of early appropriate antimicrobial therapy in Asia [23].

ARDS is one of the most common causes of severe respiratory failure that necessitates mechanical ventilation and admission to the ICU [24]. The question “What is the incidence, management and outcomes of ARDS in Asian ICUs based on the new 2023 global definitions of ARDS?” was ranked third highest for priority, reflecting interest to track both the impact of the new 2023 ARDS definitions on prevalence of ARDS and current implementation of standard ARDS management on clinical outcomes [25]. Furthermore, the concerns on validity and feasibility of the new ARDS definitions in low-resource settings have been highlighted by a recent international Delphi study [26]. Lastly, the question “What is the current practice and challenges of do not resuscitate orders, limitation of therapy and end-of-life care in Asian ICUs?” is particularly noteworthy because of the highly variable economic, cultural, and religious factors that are associated with differences in withdrawing or withholding therapies in Asian ICUs [4, 27].

Several key research priorities identified in this study are similar to those identified in other settings. A study in the UK also found that research on how to provide end-of-life care for critically ill patients and help patients recover from post-intensive syndrome was of high priority [28]. Similarly, these were also featured in a recent research priority study in low-resourced critical care settings in Asia and Africa [29]. This shows that critically ill patients and ICUs from all income settings face some common challenges despite contextual differences.

This study had several limitations. First, although we captured > 70% of the ACCCT Group members, we did not capture the entire spectrum of ICU workers in Asian ICUs as evidenced by the predominance of doctors from tertiary/teaching hospitals in this cohort. Furthermore, representation in the ACCCT group is limited to only 28 countries/regions in Asia. In the future, greater engagement with other ICU workers such as nursing, physiotherapy, and dietetic teams to facilitate multidisciplinary research and participation from non-teaching hospital ICUs across more countries and regions is needed. Second, we did not seek the opinion from critically ill patients, their families, and the wider community and other stakeholders such as administrators and policy makers in this research priority setting study. Although they are key beneficiaries, meaningful and representative views would likely have been difficult to obtain due to the range of diseases in ICU patients and differences in language, religion, and cultural background of patients in Asia. Third, we assumed that components of importance and feasibility are weighted equally in ranking research questions even though individual hospitals from different income settings may value importance and feasibility components differently. Weighted analysis was not used because this approach would still be arbitrary. Instead, we included a full list of importance and feasibility scores for reference. Fourth, we prioritized and only ranked the importance and feasibility of the top 15% of the summarized research questions by popularity. The 15% threshold was arbitrarily chosen, but nonetheless a full list of suggested research questions and their popularity is provided in Supplementary Table 5.

## Conclusion

An international collaborative research network has identified 26 of the most popular research questions in critical care to drive the research agenda in Asia for the next decade. Research questions related to sepsis and ARDS were ranked most important and feasible across the region.

## Supplementary Information

Supplementary material 1. Appendix 1 ERA-ICU Phase 2 Survey. Appendix 2 Guide to Submission of Potential Research Questions for ACCCT Group. Appendix 3 ERA-ICU Study Group Contributors.

Supplementary material 2. Supplementary Table 1 Summarized Research Questions by Themes. Supplementary Table 2 Popularity of Research Themes. Supplementary Table 3 Distribution of Popularity for Summarized Research Questions. Supplementary Table 4 Threshold to identify top research questions by popularity. Supplementary Table 5 Importance and Feasibility Scores of Top 26 Summarized Research Questions. Supplementary Table 6 Importance Criteria Component Scores. Supplementary Table 7 Feasibility Criteria Component Scores. Supplementary Figure 1 Distribution of Importance and Feasibility Likert Responses.

## Data Availability

Data generated may be shared upon reasonable request to the corresponding author and approval from ethics committee.
