# Supplementary material for: Evaluation of clinical research priorities in Asian intensive care units (ERA-ICU)
Source: J Intensive Care. 2025 Aug 27;13:47. doi: 10.1186/s40560-025-00816-9 (PMC12382108; doi:10.1186/s40560-025-00816-9)
Supplement: Supplementary file 1 — Supplementary material 1. Appendix 1 ERA-ICU Phase 2 Survey. Appendix 2 Guide to Submission of Potential Research Questions for ACCCT Group. Appendix 3 ERA-ICU Study Group Contributors. [file 40560_2025_816_MOESM1_ESM.docx]

**Appendix 1-3**

|  | **Page** |
| --- | --- |
| **Appendix 1 ERA-ICU Phase 2 Survey** | 2 |
|  |  |
| **Appendix 2 Guide to Submission of Potential Research Questions for ACCCT Group** | 3 |
|  |  |
| **Appendix 3 ERA-ICU Study Group Contributors** | 5 |

**Appendix 1 ERA-ICU Phase 2 Survey**

Demographics

1. Age
2. Sex
3. Years of Critical Care Experience
4. Region/Country
5. Hospital (Tertiary, Regional, General)
6. Hospital (Urban, Rural)
7. Hospital (Teaching/Non-teaching Hospital)
8. Number of Hospital Beds
9. Number of ICU Beds
10. Type of ICU (Medical, Surgical, Cardiothoracic, Neurosurgical, Coronary)

Research Question Suggestions:

1.

2.

3.

4.

5.

6.

7.

8.

9.

10.

**Appendix 2 Guide to Submission of Potential Research Questions for ACCCT Group**

*Background:*

- to develop a set of research questions that address the knowledge gap in clinical diagnosis and management of critically ill patients, epidemiology and pathophysiology of critical illness syndromes, staff and healthcare organizational structure in Asian ICUs
- **we invite all ACCCT Group members to submit research questions**
- submitted research questions will be grouped into themes and then ranked by National and Regional Representatives using pre-determined importance and feasibility criteria (link provided)

*Guidance:*

- research questions should have emphasis on topics important to, though not necessarily unique to, Asian ICUs
- identify relevant research questions that can be addressed from now to 2030
- should benefit ICU patients or healthcare staff
- should be important and feasible (link provided)
- questions need sufficient detail but do not need to specifically explain study design
- as reference here are some examples of research questions that guided our previous collaborative studies

|  | **Objectives:** | **Research Theme** | **Sample Research Question** |
| --- | --- | --- | --- |
| **MOSAICS II**  Li A, Ling L, Qin H, et al. Epidemiology, Management, and Outcomes of Sepsis in ICUs among Countries of Differing National Wealth across Asia. Am J Respir Crit Care Med. 2022 Nov 1;206(9):1107-1116. doi: 10.1164/rccm.202112-2743OC. | To evaluate across countries/regions of differing income status in Asia 1) the prevalence, causes, and outcomes of sepsis as a reason for ICU admission and 2) sepsis bundle (antibiotic administration, blood culture, and lactate measurement) compliance and its association with hospital mortality | Sepsis Management  Sepsis Epidemiology | How does the management of sepsis in different types of Asian ICUs compare to Surviving Sepsis Campaign recommendations?  Does compliance with sepsis management bundles improve survival?  What are the characteristics and outcomes of patients who are admitted to the ICU for sepsis? |
| **ACME**  Phua J, Joynt GM, Nishimura M, et al. Withholding and Withdrawal of Life-Sustaining Treatments in Intensive Care Units in Asia. *JAMA Intern Med.* 2015;175(3):363–371. doi:10.1001/jamainternmed.2014.7386 | To describe physicians’ attitudes toward withholding and withdrawal of life-sustaining treatments in end-of-life care and to evaluate factors associated with observed attitudes. | Ethics and end of life care | What is the current practice of withholding and withdrawal of life-sustaining treatments in Asian ICUs?  What are the factors that are associated with physician attitudes in end-of-life care? |
| **Asian ABC**  Phua J, Faruq MO, Kulkarni AP, et al. Critical Care Bed Capacity in Asian Countries and Regions. Crit Care Med. 2020 May;48(5):654-662. doi: 10.1097/CCM.0000000000004222. | To assess the number of adult critical care beds in Asian countries and regions in relation to population size. | Organization characteristics of Asian ICUs | What is the critical care bed capacity across different income countries/regions of Asia? |
| **SABA**  See KC, Zhao MY, Nakataki E, et al. Professional burnout among physicians and nurses in Asian intensive care units: a multinational survey. Intensive Care Med. 2018 Dec;44(12):2079-2090. doi: 10.1007/s00134-018-5432-1. | We investigated burnout prevalence and its associated risk factors among Asian intensive care unit (ICU) physicians and nurses. | Workforce planning and occupational health | What is the prevalence of burnout amongst Asian ICU healthcare staff?  What are the factors that are associated with higher rates of burnout in Asian ICU healthcare staff? |
| **ASIP**  Arabi YM, Phua J, Koh Y, et al. Structure, Organization, and Delivery of Critical Care in Asian ICUs. Crit Care Med. 2016 Oct;44(10):e940-8. doi: 10.1097/CCM.0000000000001854. | To describe the structure, organization, and delivery in Asian ICUs. | Organization characteristics of Asian ICUs | What is the staffing level and number of ICU beds across different income countries/regions of Asia?  What are the facilities for single room and negative pressure room isolation in Asian ICUs? |

**Appendix 3** **ERA-ICU Study Group Contributors**

*National/Regional Representative

**Bangladesh**

| **Investigator** | **Hospital** |
| --- | --- |
| Ariful Basher | Infectious Diseases Hospital |
| Mahbub Morshed | Dinajpur Medical College Hospital |
| Mohammad Omar Faruq* | United Hospital |
| Tasmia Kashfi | United Hospital |

**Brunei**

| **Investigator** | **Hospital** |
| --- | --- |
| Khalid Mahmood Khan Nafees* | Raja Isteri Pengiran Anak Saleha Hospital |

**China**

| **Investigator** | **Hospital** |
| --- | --- |
| Bin Lin | Changxing People's Hospital |
| Chun Pan | Sichuan Provincial People's Hospital |
| Dechang Chen | Shanghai Ruijin Hospital |
| Gang Wang | The Second Affiliated Hospital of Xi'an Jiaotong University |
| Jiao Liu | Shanghai Ruijin Hospital |
| Lihui Wang | Renji Hospital, Shanghai Jiao Tong University, School of Medicine |
| Ling-Ai Pan | Sichuan Provincial People's Hospital |
| Lining Si | Qinghai University Affiliated Hospital |
| Xu Wei | Chengdu Seventh People's Hospital |
| Yi Zhang | Sichuan Provincial People's Hospital |
| Yiping Wang | Sichuan Provincial People's Hospital |
| Yuetian Yu | Renji Hospital, Shanghai Jiao Tong University, School of Medicine |
| Zhongheng Zhang* | Sir Run Run Shaw Hospital |

**Hong Kong SAR, China**

| **Investigator** | **Hospital** |
| --- | --- |
| Hoi Ping Shum | Pamela Youde Nethersole Eastern Hospital |
| Kwok-Ming Ho | Prince of Wales Hospital |
| Lowell Ling | Prince of Wales Hospital |
| Pauline Yeung Ng* | Queen Mary Hospital |
| Man Yee Man | Pamela Youde Nethersole Eastern Hospital |
| Wai-Tat Wong | Prince of Wales Hospital |

**India**

| **Investigator** | **Hospital** |
| --- | --- |
| Anirban Hom Choudhuri | Safdarjung Hospital and Vardhman Medical College |
| Badri Prasad Das | Institute of Medical Sciences, Banaras Hindu University |
| Bharat G Jagiasi | Kokilaben Dhirubhai Ambani Hospital |
| Deepak Govil | Medantas The Medicity |
| Jigeeshu Divatia | Lilavati Hospital and Research Centre |
| Mehul Shah | Sir H N Reliance Foundation and Research Hospital |
| Pravin Amin | Bombay Hospital Institute of Medical Sciences |
| Rajesh Mishra | Epic Hospital |
| Rajesh Mohan Shetty | Manipal Hospital Whitefield |
| Ravi Shankar | KIMS Saveera Hospital |
| Sheila Nainan Myatra* | Tata Memorial Hospital |
| Shivangi Mishra | Manipal Hospital Whitefield |
| Simant Jha | Pushpawati Singhania Research Institute Hospital |
| Subhal Dixit | Sanjeevan Hospital |
| Swarna Deepak Kuragayala | Apollo Health City |
| R Vaidyanathan | Cauvery Heart and Multi-specialty Hospital |
| Ziyokov Joshi | Tagore Hospital and Heart Care Centre |

**Indonesia**

| **Investigator** | **Hospital** |
| --- | --- |
| Erwin Pradian | Hasan Sadikin Hospital |
| Faisal Muchtar* | Wahidin Sudirohusodo Hospital |
| Haizah Nurdin | Wahidin Sudirohusodo Hospital |
| Nurita Dian Kestriani Saragi Sitio | Hasan Sadikin Hospital |

**Iran**

| **Investigator** | **Hospital** |
| --- | --- |
| Seyed Mohammad Reza Hashemian* | Masih Daneshvari Hospital |

**Japan**

| **Investigator** | **Hospital** |
| --- | --- |
| Hideto Yasuda | Jichi Medical University Saitama Medical Center |
| Kazuaki Atagi | Nara Prefectural General Medical Center |
| Moritoki Egi* | Kyoto University Hospital |

**Kazakhstan**

| **Investigator** | **Hospital** |
| --- | --- |
| Aidos Konkayev* | National Scientific Center of Traumatology and Orthopedics |
| Benazir Azimova | National Scientific Center of Traumatology and Orthopedics |
| Maiya Konkayeva | Municipal Infection Hospital |
| Nursultan Dauletbaev | National Scientific Center of Traumatology and Orthopedics |

**Kuwait**

| **Investigator** | **Hospital** |
| --- | --- |
| Abdulrahman Al-Fares* | Al-Amiri Hospital |
| Dalal Aldosari | Al-Amiri Hospital |
| Sajida Sange | Al-Amiri Hospital |

**Lebanon**

| **Investigator** | **Hospital** |
| --- | --- |
| Carine Harmouche* | Hotel-Dieu de France |
| Zeina Aoun Bacha | Hotel-Dieu de France |

**Malaysia**

| **Investigator** | **Hospital** |
| --- | --- |
| Ji Zhang Chin | Hospital Tuanku Ja'afar Seremban |
| M. Shanaz Hasan | University of Malaya Medical Centre |
| Mohd Basri Mat Nor* | Sultan Ahmad Shah Medical Centre @IIUM |
| Muhamad Hafizzi | Melaka General Hospital |
| Nor’azim Mohd Yunos | University of Malaya Medical Centre |
| Zheng-Yii Lee | University of Malaya Medical Centre |

**Mongolia**

| **Investigator** | **Hospital** |
| --- | --- |
| Enkhsaikhan Samdan | The Third State Central Hospital |
| Erdenechimeg Tegshee | The Third State Central Hospital |
| Mendsaikhan Naranpurev* | Mongolia Japan Hospital |
| Suvd-Erdene Narmandakh | Mongolia Japan Hospital |
| Tamir Lkhagvadorj | Mongolia Japan Hospital |
| Telmen Amartur | Mongolia-Japan Hospital |
| Tsolmon Begzjav | Intermed Hospital |

**Nepal**

| **Investigator** | **Hospital** |
| --- | --- |
| Gentle Sunder Shrestha* | Tribhuvan University Teaching Hospital |

**Oman**

| **Investigator** | **Hospital** |
| --- | --- |
| Mahir Al Bahrani | Royal Hospital |

**Pakistan**

| **Investigator** | **Hospital** |
| --- | --- |
| Ashok Kumar | Doctor Ziauddin University Hospital |
| Muhammad Sohaib | Aga Khan University |
| Muneeb Ali | Pakistan Institute of Medical Sciences |
| Sheharyar Ashraf | Lady Reading Hospital |

**Philippines**

| **Investigator** | **Hospital** |
| --- | --- |
| Debbie Noblezada-Uy | The Medical City Iloilo Hospital |
| Faith Joan Gaerlan | Southern Philippines Medical Center |
| Gerardo Briones, Jr | Asian Hospital And Medical Center |
| Jeremiah Butch T. Gemarino | Adventist Hospital Palawan and Philippine Heart Center |
| Jose Emmanuel Palo* | The Medical City |
| Kevin De Asis | St Luke's Medical Center |
| Marion Patricio | Philippine General Hospital |
| Pauline Convocar | Manila Doctors Hospital |
| Rodolfo Roman Bigornia | Chong Hua Hospital and Cebu Doctors' University Hospital |

**Republic of Korea**

| **Investigator** | **Hospital** |
| --- | --- |
| Byunghyuk Yu | Kyungpook National University Chilgok Hospital |
| Jae Kyeom Sim | Korea University Guro Hospital |
| Jongmin Lee | Seoul St. Mary's Hospital |
| Jung-Min Bae | Yeungnam University Medical Center |
| Kyoung Hoon Lim | Kyungpook National University Hospital |
| Kyungsoo Chung | Severance Hospital |
| Soyoung Park | Eunpyeong St. Mary's Hospital |
| Sejoong Kim | Seoul National University Bundang Hospital |
| Song I Lee | Chungnam National University Hospital |
| Won-Young Kim | Chung-Ang University Hospital |
| Young-Jae Cho* | Seoul National University Bundang Hospital |

**Saudi Arabia**

| **Investigator** | **Hospital** |
| --- | --- |
| Abdullah M. Alhammad | King Saud University |
| Ayman Kharaba* | King Fahad Hospital |
| Carlos Sanchez | King Faisal Specialist Hospital and Research Centre |
| Faten Farid Awdallah | King Abdullah Medical City |
| Ghaleb Almekhlafi | King Salman Bin Abdulaziz Medical City |
| Mohamed Hegazy | Ministry of National Guard Health Affairs Hospital |
| Mohammed Alshahrani | King Fahad Hospital of the University |
| Rawah Shafiq Aljishi | King Salman Hospital |
| Samiyah Alanazi | Ministry of National Guard Health Affairs Hospital |
| Yaseen M. Arabi | King Abdulaziz Medical City |
| Zainab Al Duhailib | King Faisal Specialist Hospital and Research Centre |

**Singapore**

| **Investigator** | **Hospital** |
| --- | --- |
| Amartya Mukhopadhyay | National University Hospital |
| Andrew Li* | Woodlands Hospital |
| Balachandran Kayachandran | National University Hospital |
| Charles Chin Han Lew | Ng Teng Fong General Hospital |
| Clarabella Liew | Tan Tock Seng Hospital |
| Geetha Kayambu | National University Hospital |
| Jason Phua | Alexandra Hospital |
| John Tsoong | National University Hospital |
| K Ramanathan | National University Hospital |
| Kay Choong See | National University Hospital |
| Kumaresh Venkatesan | Khoo Teck Puat Hospital |
| Matthew Cove | National University Hospital |
| Roshni Gokhale | Changi General Hospital |
| Sharlene Ho | Tan Tock Seng Hospital |
| Shir Lynn Lim | National University Heart Centre Singapore |
| Yew Woon Chia | Tan Tock Seng Hospital |
| Yi Hern Tan | Singapore General Hospital |
| Yie Hui Lau | Tan Tock Seng Hospital |
| Yu-Lin Wong | Tan Tock Seng Hospital |
| Will Loh | National University Hospital |

**Taiwan**

| **Investigator** | **Hospital** |
| --- | --- |
| Cong-Tat Cia | National Cheng Kung University Hospital |
| Han-Chung Hu | Chang Gung Memorial Hospital |
| Jia-Jun Wu | Chung Shan Medical University Hospital |
| Jia-Yih Feng | Taipei Veterans General Hospital |
| Ming-Cheng Chan* | Taichung Veterans General Hospital |
| Ming-Chieh Yang | E-Da Cancer Hospital |
| Sheng-Yuan Ruan | National Taiwan University Hospital |

**Turkey**

| **Investigator** | **Hospital** |
| --- | --- |
| Mehmet Uyar* | Ege University Hospital |

**United Arab Emirates**

| **Investigator** | **Hospital** |
| --- | --- |
| Alaaeiden Ghanem | Sheikh Khalifa Medical City |
| Imadeddin Barakat | Danat Al Emarat Hospital |
| Jihad Mallat | Cleveland Clinic Abu Dhabi |
| Prashant Nasa | NMC Specialty Hospital |
| Rania Omar | Ibrahim Bin Hamad Obaidallah Hospital |
| Reda Mohamed Sherif | Kuwait Hospital |

**Vietnam**

| **Investigator** | **Hospital** |
| --- | --- |
| Binh Son Ha | Da Nang Hospital |
| Chinh Quoc Luong | Bach Mai Hospital |
| Dai Quang Huynh | Cho Ray Hospital |
| Hieu Huu Hoang | Da Nang Hospital |
| Huan Huu Nguyen | Argriculture General Hospital |
| Hung Ngoc Dinh | Phu Tho Provincial General Hospital |
| Hung Tan Nguyen | Da Nang Hospital |
| Minh Hoa Le | Da Nang Hospital |
| Kien Trung Nguyen | Military Medical Department |
| Phuoc Thien Duong | Can Tho Central General Hospital |
| Son Ngoc Do* | Bach Mai Hospital |
| Dung Tat Nguyen | Hue Central General Hospital |
| Thai Van Hoang | Nghe An Provincial Friendship General Hospital |
| Thao Thi Ngoc Pham | Cho Ray Hospital |
| Thien Xuan Mai | Vinmec Times City International Hospital |
| Thuy Thi Phuong Le | Dong Da General Hospital |
| To Dang Nguyen | Phu Tho Provincial General Hospital |
| Chinh Huy Vu | Phuc Yen Regional General Hospital |
