# Supplementary material for: Evaluation of clinical research priorities in Asian intensive care units (ERA-ICU)
Source: J Intensive Care. 2025 Aug 27;13:47. doi: 10.1186/s40560-025-00816-9 (PMC12382108; doi:10.1186/s40560-025-00816-9)
Supplement: Supplementary file 2 — Supplementary material 2. Supplementary Table 1 Summarized Research Questions by Themes. Supplementary Table 2 Popularity of Research Themes. Supplementary Table 3 Distribution of Popularity for Summarized Research Questions. Supplementary Table 4 Threshold to identify top research questions by popularity. Supplementary Table 5 Importance and Feasibility Scores of Top 26 Summarized Research Questions. Supplementary Table 6 Importance Criteria Component Scores. Supplementary Table 7 Feasibility Criteria Component Scores. Supplementary Figure 1 Distribution of Importance and Feasibility Likert Responses. [file 40560_2025_816_MOESM2_ESM.docx]

|  | **Page** |
| --- | --- |
| **Supplementary Table 1 Summarized Research Questions by Themes** | 2 |
|  |  |
| **Supplementary Table 2 Popularity of Research Themes** | 9 |
|  |  |
| **Supplementary Table 3 Distribution of Popularity for Summarized Research Questions** | 10 |
|  |  |
| **Supplementary Table 4 Threshold to identify top research questions by popularity** | 11 |
|  |  |
| **Supplementary Table 5 Importance and Feasibility Scores of Top 26 Summarized Research Questions** | 12 |
|  |  |
| **Supplementary Table 6 Importance Criteria Component Scores** | 14 |
|  |  |
| **Supplementary Table 7 Feasibility Criteria Component Scores** | 16 |
|  |  |
| **Supplementary Figure 1 Distribution of Importance and Feasibility Likert Responses** | 18 |

**Supplementary Tables and Figures**

**Supplementary Table 1 Summarized Research Questions by Themes**

| **Theme** | **Question** | **Popularity** |
| --- | --- | --- |
| AKI/Renal Replacement Therapy | What is the incidence, pathophysiology and outcomes of AKI in Asian ICUs? | 6 |
|  | What is the optimal timing of initiation of renal replacement therapy for AKI? | 3 |
|  | What are the current practices of kidney replacement therapy and their impact on outcomes in Asian Intensive Care Units? | 1 |
|  | What are the ways to predict, prevent and improve outcomes of AKI? | 3 |
|  | What are the risk factors for therapeutic failure of renal replacement therapy? | 1 |
| Cardiology/Cardiothoracic/Cardiac Arrest | What is the prevalence, characteristics, management and outcomes of resuscitated out-of-hospital cardiac arrest in Asian ICUs? | 3 |
|  | What is the current practice and cost-effectiveness of post-cardiac arrest care and advanced neuro-prognostication in Asian ICUs across all income settings? | 4 |
|  | Does protocolized post-cardiac arrest care improve outcomes? | 1 |
|  | What is the structure, organization, and delivery of cardiac critical care in Asian ICUs? | 3 |
|  | What is the prevalence, management and outcomes of acute heart failure? | 2 |
|  | Can preventive therapy reduce risk of acute heart failure decompensation in patients with dilated cardiomyopathy? | 1 |
|  | What is the prevalence, management and outcomes of pulmonary hypertension in Asian ICUs? | 2 |
|  | What is the optimal use of inotropes and vasopressors in septic shock with septic cardiomyopathy? | 1 |
|  | What are the factors associated with survival after STEMI without revascularization in resource limited settings? | 1 |
|  | What are the outcomes of cardiac surgery in Asian ICUs? | 1 |
| Delirium/Sedation | What pharmacological and non-pharmacological strategies can prevent and treat delirium in ICU patients? | 5 |
|  | What is the incidence of delirium and current practice on delirium identification, prevention and treatment in Asian ICUs? | 4 |
|  | What is the optimal use of sedation in the ICU? | 6 |
| ECLS | What is the capacity and role of ECLS in different income and resource settings? | 3 |
|  | What is the optimal anticoagulation target in Asian patients on ECLS? | 1 |
|  | What is the role of ECLS in ARDS and COPD in Asian ICUs? | 2 |
|  | What is the incidence, characteristics and outcomes of patients on ECLS in Asian ICUs? | 3 |
|  | Does prone positioning improve outcomes in patients on VV-ECMO in Asian ICUs? | 1 |
|  | What are the outcomes of immunocompromised patients on ECLS in Asian ICUs? | 1 |
|  | How is platelet function in patients on ECLS best assessed? | 1 |
| Family | Does family engagement improve patient outcomes and relationship between ICU team and patient and/or relatives ? | 2 |
|  | What is the visiting arrangement and role of family members in decision making in Asian ICUs? | 2 |
| General ICU Care | Can artificial intelligence predict and improve outcomes of ICU patients across all income settings? | 5 |
|  | What are the transfusion targets and practices in Asian ICUs? | 2 |
|  | What blood transfusion strategies can improve outcomes of ICU patients? | 1 |
|  | What is the optimal way to control blood sugar in Asian ICUs? | 1 |
|  | How does diabetes and baseline HbA1c affect outcome prediction and management of ICU patients? | 2 |
|  | What are the characteristics and outcomes of elderly patients admitted to Asian ICUs? | 3 |
|  | Is existing formulas for predicted body weight valid in Asian population? | 1 |
|  | How does obesity affect outcome of ICU patients? | 2 |
|  | What are the clinical factors associated with outcomes in Asian ICUs? | 1 |
|  | How are triage decisions for admission made in Asian ICUs? | 1 |
|  | How does social determinants of health affect incidence and outcomes of critical illness in Asia? | 1 |
|  | What is the impact of prolonged ICU stay on quality of life and survival in Asian ICUs? | 1 |
|  | What is the role of social workers in Asian ICUs? | 1 |
|  | What are the characteristics and outcomes of critically ill patients managed in the emergency department? | 1 |
|  | What is the epidemiology of autoimmune diseases in Asian ICUs? | 1 |
|  | How to improve sustainability and environmental awareness in Asian ICUs? | 2 |
|  | How to deliver cost-effective critical care services in Asian ICUs? | 1 |
|  | Does protocolized standard routine care in ICU improve patient outcomes? | 2 |
|  | Does selective digestive decontamination work in Asian ICUs? | 2 |
|  | What is the optimal use of stress ulcer prophylaxis? | 3 |
|  | What is the prevalence, characteristics and outcomes of acute on chronic liver failure in Asian ICUs? | 3 |
|  | Does double plasma molecular adsorption system improve outcomes for patients with liver failure? | 1 |
|  | What is the role of glucocorticosteroid in critical illness? | 2 |
|  | What are the outcomes of immunocompromised patients with respiratory failure in Asian ICUs? | 1 |
|  | What is the common practice and management of acute poisoning in Asian ICUs? | 2 |
|  | What is the burden of critical illness in Asia Pacific region? | 1 |
|  | What is the prevalence and impact of ICU readmission in Asia? | 3 |
|  | What are the optimal coagulation thresholds for safe insertion of central intravenous access? | 1 |
|  | What are the complications of central intravenous access? | 1 |
|  | What is the current practice of venous thromboembolism prophylaxis in Asian ICUs? | 1 |
|  | What is the prevalence and risk factors for venous thromboembolism in Asian ICUs? | 4 |
|  | What management strategies should be used for massive and submassive pulmonary embolism? | 1 |
| Infection/Sepsis | What is the current practice and challenges of antibiotic therapy in Asian ICUs? | 4 |
|  | What is the optimal way to select, initiate, dose, administer and stop antibiotic therapy in Asian ICUs? | 16 |
|  | How does antibiotic therapy differ in treatment of multidrug resistant infections in the ICU? | 3 |
|  | What the is prevalence, resistance pattern, management and outcomes of invasive fungal sepsis in Asian ICUs? | 7 |
|  | How to optimize antifungal therapy initiation, choice and duration for invasive fungal infection? | 1 |
|  | What is the pathophysiology and outcomes HIV associated critical illness? | 1 |
|  | What adjunctive treatments can shorten duration of septic shock and improve survival? | 4 |
|  | Are anti-inflammatory drugs and immune modulator useful in sepsis? | 5 |
|  | What are the long term cognitive, physical, psychological effects of sepsis? | 2 |
|  | What is the prevalence and outcomes of multidrug resistant infections and sepsis in Asian ICUs? | 17 |
|  | What are the genetic variants that are associated with mortality in sepsis patients of Asian descent? | 1 |
|  | What is the role of extracorporeal purification or plasma exchange in sepsis? | 5 |
|  | What are the molecular and clinical phenotypes of sepsis? | 2 |
|  | What biomarkers can be used for early diagnosis of sepsis? | 2 |
|  | What is the role of routine surveillance cultures in diagnosing ICU acquired infections? | 2 |
|  | What is the prevalence, characteristics and outcomes of nosocomial infections in Asian ICUs? | 3 |
|  | What are the barriers to good sepsis care and bundle compliance and can they be improved? | 3 |
|  | What is the compliance rate and associated outcomes of sepsis management bundles in Asian ICUs? | 1 |
|  | What are the strategies to prevent nosocomial infection and sepsis in Asian ICUs? | 3 |
|  | What is the incidence and mortality of sepsis and septic shock in Asia? | 6 |
|  | How is sepsis diagnosed in Asian ICUs and what are the factors associated with early detection? | 6 |
|  | Can clinical predictive scores predict sepsis mortality? | 1 |
|  | What is the prevalence, characteristics and outcomes of sepsis from non-bacterial and tropical infections in Asian ICUs? | 2 |
|  | What is the current practice on antibiotic therapy, antibiotic stewardship and antimicrobial resistance in sepsis and septic shock in Asian ICUs? | 3 |
|  | What is the role of rapid molecular pathogen detection in Asian ICUs? | 2 |
|  | What is the optimal fluid resuscitation strategy for septic shock across different income settings? | 3 |
|  | Do safety checklists improve diagnosis and management of sepsis? | 1 |
|  | How to improve the outcome of bacterial community acquired pneumonia? | 1 |
|  | What are the common pathogens in severe community acquired pneumonia in Asian ICUs? | 2 |
|  | Does personalized hemodynamic targets in septic shock improve outcomes? | 2 |
|  | What is the impact of screening for sepsis in non-critical care settings? | 1 |
|  | What is the prevalence of asymptomatic carriage of resistant bacterial pathogens amongst ICU staff and is this associated with patient outcomes? | 1 |
|  | What is the prevalence and outcomes of thrombocytopenia in critically ill patients with sepsis? | 1 |
|  | What is the best way to assess immune function in patients with sepsis? | 2 |
|  | What is the effect of anaemia on development of prosthetic infections? | 1 |
|  | How does endothelial dysfunction affect resuscitation in septic shock? | 1 |
|  | Does a quality improvement programme aimed at improving specific aspects of essential emergency and critical care improve outcomes of patients with sepsis? | 1 |
|  | What are the mechanical ventilation strategies for patients with respiratory failure from pulmonary tuberculosis? | 1 |
|  | What should be the standards for management of patients with severe dengue? | 2 |
|  | What is the epidemiology, management and outcomes of TB in Asian ICUs? | 1 |
| Neurology/Neurocritical Care | What is the best scoring system to monitor neurological status in Asian ICUs? | 1 |
|  | What is the outcome of critically ill patients with stroke in low resource settings? | 1 |
|  | Does specialized stroke critical care units improve outcomes of patients with stroke? | 1 |
|  | What drugs can improve outcomes of patients with subarachnoid hemorrhage? | 1 |
|  | Does perfusion scan improve patient selection for intra-arterial thrombolysis? | 1 |
|  | What routine care should be provided for patients with reduced consciousness? | 1 |
|  | What are the ways to improve neurological recovery of stroke? | 1 |
|  | What is the best non-invasive method to measure intracranial pressure? | 1 |
| Nutrition/Rehabilitation | Does early enteral nutrition improve outcomes in severe Pancreatitis? | 1 |
|  | Does administration of protein supplementation based on muscle mass improve functional outcomes in critically ill patients? | 1 |
|  | What is the prevalence of malnutrition and nutritional deficit and current nutritional practices (including staffing support) in Asian ICUs? | 2 |
|  | Can indirect calorimetry improve patient outcomes in Asian ICUs? | 1 |
|  | What is the rate of diaphragmatic muscle wasting in ICU patients and its association with protein intake? | 1 |
|  | Does tailoring energy and protein dose with daily clinical severity status improve functional and mortality in critically ill patients? | 1 |
|  | What is the relationship between energy expenditure and nutrition and exercise requirements in critically ill patients? | 2 |
|  | What is the prevalence, management and outcomes of post-intensive care syndrome? | 6 |
|  | Can early mobilization preserve muscle mass, improve biomarkers and clinical outcomes in Asian ICUs? | 4 |
|  | What is the role of rehabilitation centers and post-ICU clinics in Asian ICUs? | 4 |
|  | What strategies can help prevent ICU acquired weakness? | 3 |
|  | Can MRC score and muscle mass be used to objectively assess ICU acquired weakness? | 1 |
|  | Can needs for rehabilitation and length of stay be predicted? | 1 |
| Palliative Care | What is the current practice and challenges of do not resuscitate orders, limitation of therapy and end of life care in Asian ICUs? | 8 |
|  | Can understanding of socioeconomic, religious and cultural factors improve end of life decisions in Asian ICUs? | 3 |
|  | What is the prevalence and outcomes of admission for palliative care in Asian ICUs? | 1 |
|  | What is the prevalence and outcomes of patients admitted for time limited trials in Asian ICUs? | 1 |
| Perioperative Care/Trauma | Does multidisciplinary approach improve perioperative outcomes in Asian ICUs? | 1 |
|  | What are the factors that optimize damage control surgery in trauma? | 1 |
|  | Can a predictive model be used to model outcomes of traumatic brain injury in Asia? | 1 |
|  | What are the outcomes of patients with traumatic brain injury in Asian ICUs? | 1 |
|  | What are the ways to improve neurological recovery in traumatic brain injury? | 3 |
|  | What is the best way to control neurogenic fever after traumatic brain injury? | 1 |
|  | What are the clinical and epidemiologic factors that can reduce mortality amongst patients with trauma? | 1 |
|  | What is the cost-effectiveness of trauma team activation for a critically ill patients due to polytrauma? | 1 |
|  | What is the role for pre-hospital tranexamic acid in severe trauma in regions where there are no pre-hospital trauma response systems? | 1 |
| Respiratory Care/Failure | What are the genotypes, phenotypes and pathophysiology of ARDS? | 3 |
|  | Can precision medicine be used to predict and improve outcomes of ARDS? | 2 |
|  | What is the incidence, management and outcomes of ARDS in Asian ICUs based on the new 2023 global definitions of ARDS? | 6 |
|  | What respiratory and ventilator strategies can improve outcomes of ARDS? | 2 |
|  | Can anti-inflammatory agents prevent and treat ARDS? | 2 |
|  | What is the current practice and rates of liberation from mechanical ventilation in Asian ICUs? | 2 |
|  | What is the impact of high flow nasal oxygen devices and non-invasive ventilation on outcomes across different income settings? | 2 |
|  | What are current practices on use of high flow nasal oxygen devices and non-invasive ventilation in Asian ICUs? | 1 |
|  | What is the role of high flow nasal oxygen during tracheal intubation? | 1 |
|  | Does personalized oxygen targets before and during ICU care improve patient outcomes? | 2 |
|  | What are the ways of improving PEEP titration in critically ill patients? | 2 |
|  | What is the current practice on use of neuromuscular blockade in Asian ICUs? | 1 |
|  | What is the adherence and satisfaction of the medical staff to protocolized supplemental oxygen use? | 1 |
|  | What is the optimal way to transport patients on high flow oxygen who does not require immediate mechanical ventilation? | 1 |
|  | What is the role of high flow nasal oxygen and non-invasive ventilation in patients with acute respiratory failure with shock? | 1 |
|  | What is the role of biofeedback using sensors over chest to wean patient from ventilator? | 1 |
|  | Does multidisciplinary approach improve rehabilitation in mechanically ventilated patients? | 1 |
|  | What are the methods to monitor respiratory mechanics? | 1 |
|  | What positions and ventilator settings improve perfusion in ARDS? | 1 |
|  | Does high flow oxygen shorten liberation from mechanical ventilation in tracheostomized patients? | 1 |
|  | What are the optimal analgesics in patients on mechanical ventilation? | 1 |
|  | What is the epidemiology of acute respiratory failure in Asian ICUs? | 2 |
|  | How is acute respiratory failure best assessed? | 1 |
|  | What is the current practice of approaches to tracheostomy in Asian ICUs? | 1 |
| Shock/Intravenous Fluids | What is the optimal maintenance intravenous fluids to use in Asian ICUs? | 3 |
|  | What is the current practice of intravenous fluids usage in Asian ICUs? | 2 |
|  | What is the role of albumin in different types of critical illness? | 2 |
|  | What are the best techniques and devices to assess fluid requirement in Asian ICUs? | 4 |
|  | What is the optimal timing, dose and duration of vasopressors in shock? | 1 |
|  | Is peripheral noradrenaline safe in the non-critical care setting? | 1 |
|  | When should vasopressin be used in septic shock? | 1 |
|  | Does adjunctive hydrocortisone in acute myocardial prevent cardiogenic shock? | 1 |
|  | What is the role of vasopressin in cardiogenic shock? | 2 |
| Structure/Training/Staffing/Teamwork/Safety | What are the challenges in communication with other specialty and teams in Asian ICUs? | 1 |
|  | What is the role of handover when critically ill patients are transferred from different stages of care (prehospital, emergency department, ward, ICU)? | 1 |
|  | How are complaints and medical errors handled in Asian ICUs? | 1 |
|  | What is the role of psychiatrist in Asian ICUs? | 1 |
|  | What are steps needed to expand the functions of Asian Critical Care Societies? | 1 |
|  | What is the prevalence and factors associated with burn out amongst healthcare staff in Asian ICUs? | 5 |
|  | What is the current and optimal staff to patient ratio and type of training across Asian ICUs from different income settings and types of ICU? | 10 |
|  | What factors are associated with staff well being in Asian ICUs? | 1 |
|  | Does burnout amongst staff affect patient outcomes? | 1 |
|  | What is the current role and staffing of clinical pharmacists in Asian ICUs? | 2 |
|  | What strategies can reduce burnout amongst ICU staff? | 1 |
|  | What is the impact of staff to patient ratio on patient centered outcomes? | 1 |
|  | How is workload related to stress and burnout amongst ICU staff? | 1 |
|  | How does organization factors such as referral system, open/closed, ICU/hospital bed capacity affect patient outcomes in Asian ICUs? | 6 |
|  | What are the population ICU bed capacities across Asia? | 1 |
|  | What is the effect of triage on patient outcomes? | 1 |
|  | What is the respiratory therapist and ventilated patient ratio across Asian ICUs? | 1 |
|  | What is considered basic competence of nursing staff in the ICU? | 1 |
|  | What are the factors that delay ICU admission/discharge in Asia? | 1 |
|  | What are the current practices of high-dependency and step-down units in Asian countries? | 1 |
|  | What are the gaps in the provision of critical care in low and middle-income countries compared to high-income countries, and in rural areas compared to urban areas, in Asia? | 1 |
|  | What are the barriers and facilitators to the adoption of tele-ICU services in enhancing the management of critically ill patients in Asian countries with varying levels of healthcare infrastructure? | 1 |
|  | What are the challenges for small ICUs in non-tertiary hospitals and tertiary referral systems in Asia? | 1 |
|  | What is the burden of bedside procedures (bedside tracheostomy, bedside upper and lower endoscopy) in Asian ICUs? | 1 |
|  | What are the ICU admission and discharge criteria used in Asian ICUs across different income settings? | 1 |
|  | What are the staffing patterns and other organizational factors in hospital areas outside the ICU which also manage critically ill patients? | 1 |
|  | What is the economic burden of critical care in the emergency department? | 1 |
| Ultrasound | What are the available training schemes for implementing a standardised POCUS among different Asian ICUs? | 1 |
|  | Can point of care ultrasound improve diagnosis and management of critically ill patients? | 5 |
|  | What is the best way to teach point of care ultrasound in critical care? | 1 |
|  | What is the current utilization of point of care ultrasound in Asian ICUs? | 2 |

List of 197 summary research questions generated from 408 submitted research question suggestions collected in Phase 2. Questions were categorized into 15 research themes. The number of mentions for each summarized research question was calculated by counting the number of times the question was mentioned in the original list of submitted research questions. AKI, acute kidney injury; ARDS, acute respiratory distress syndrome; COPD, chronic obstructive pulmonary disease; ECLS, extracorporeal life support; ECMO, extracorporeal membrane oxygenation; HIV, human immunodeficiency virus; ICU, intensive care unit; PEEP, positive end expiration pressure; POCUS, point of care ultrasound; STEMI, ST-elevation myocardial infarction; TB, tuberculosis.

**Supplementary Table 2 Popularity of Research Themes**

| **Theme** | **Number of Mentions (%)** |
| --- | --- |
| AKI/Renal Replacement Therapy | 14 (3.4) |
| Cardiology/Cardiothoracic/Cardiac Arrest | 19 (4.6) |
| Delirium/Sedation | 15 (3.6) |
| ECLS | 12 (2.9) |
| Family | 4 (1.0) |
| General ICU Care | 55 (13.3) |
| Infection/Sepsis | 123 (30.0) |
| Neurology/Neurocritical Care | 8 (1.9) |
| Nutrition/Rehabilitation | 28 (6.8) |
| Palliative Care | 13 (3.1) |
| Perioperative Care/Trauma | 11 (2.7) |
| Respiratory Care/Failure | 39 (9.4) |
| Shock/Intravenous Fluids | 17 (4.1) |
| Structure/Training/Staffing/Teamwork/Safety | 46 (11.1) |
| Ultrasound | 9 (2.2) |

The popularity of research themes according to number of mentions accumulated by 197 summarized research questions from the 408 original research question suggestions in Phase 2. AKI, acute kidney injury; ECLS, extracorporeal life support; ICU, intensive care unit.

**Supplementary Table 3 Distribution of Popularity for Summarized Research Questions**

| **Mentions** | **Number of Questions** |
| --- | --- |
| 1 | 112 |
| 2 | 38 |
| 3 | 21 |
| 4 | 8 |
| 5 | 6 |
| 6 | 7 |
| 7 | 1 |
| 8 | 1 |
| 10 | 1 |
| 16 | 1 |
| 17 | 1 |

The distribution for number of mentions gathered by 197 summarized research questions from 408 original research question suggestions in Phase 2.

**Supplementary Table 4 Threshold to identify top research questions by popularity**

| **Threshold (number of mentions)** | **Number of Questions Identified Using Threshold** | **Representation of Top Research Questions by Popularity** |
| --- | --- | --- |
| 1 | 197 | 100% |
| 2 | 85 | 43% |
| 3 | 47 | 24% |
| 4 | 26 | 13% |
| 5 | 18 | 9% |
| 6 | 12 | 6% |
| 7 | 5 | 3% |
| 8 | 4 | 2% |
| 10 | 3 | 2% |
| 16 | 1 | 1% |
| 17 | 1 | 1% |

Number of questions and representation of top research questions by popularity (%) when different thresholds of number of mentions are used. These 197 summarized research questions were generated from 408 original research question suggestions in Phase 2.

**Supplementary Table 5 Importance and Feasibility Scores of Top 26 Summarized Research Questions**

| **Theme** | **Question** | **Priority**  **Rank** | **Importance** | **Feasibility** | **Total** | **Popularity** |
| --- | --- | --- | --- | --- | --- | --- |
| AKI/Renal Replacement Therapy | What is the incidence, pathophysiology and outcomes of AKI in Asian ICUs? | 13 | 514 | 518 | 1032 | 6 |
| Cardiology/Cardiothoracic/Cardiac Arrest | What is the current practice and cost-effectiveness of post-cardiac arrest care and advanced neuro-prognostication in Asian ICUs across all income settings? | 15 | 498 | 499 | 997 | 4 |
| Delirium/Sedation | What is the optimal use of sedation in the ICU? | 16 | 493 | 509 | 1002 | 6 |
|  | What pharmacological and non-pharmacological strategies can prevent and treat delirium in ICU patients? | 17 | 495 | 502 | 997 | 5 |
|  | What is the incidence of delirium and current practice on delirium identification, prevention and treatment in Asian ICUs? | 11 | 499 | 529 | 1028 | 4 |
| General ICU Care | Can artificial intelligence predict and improve outcomes of ICU patients across all income settings? | 26 | 494 | 446 | 940 | 5 |
|  | What is the prevalence and risk factors for venous thromboembolism in Asian ICUs? | 21 | 479 | 499 | 978 | 4 |
| Infection/Sepsis | What is the prevalence and outcomes of multidrug resistant infections and sepsis in Asian ICUs? | 1 | 577 | 546 | 1124 | 17 |
|  | What is the optimal way to select, initiate, dose, administer and stop antibiotic therapy in Asian ICUs? | 5 | 537 | 540 | 1077 | 16 |
|  | What the is prevalence, resistance pattern, management and outcomes of invasive fungal sepsis in Asian ICUs? | 7 | 541 | 516 | 1057 | 7 |
|  | What is the incidence and mortality of sepsis and septic shock in Asia? | 2 | 539 | 559 | 1098 | 6 |
|  | How is sepsis diagnosed in Asian ICUs and what are the factors associated with early detection? | 10 | 526 | 521 | 1046 | 6 |
|  | Are anti-inflammatory drugs and immune modulator useful in sepsis? | 18 | 461 | 490 | 951 | 5 |
|  | What is the role of extracorporeal purification or plasma exchange in sepsis? | 24 | 485 | 455 | 940 | 5 |
|  | What is the current practice and challenges of antibiotic therapy in Asian ICUs? | 9 | 520 | 536 | 1056 | 4 |
|  | What adjunctive treatments can shorten duration of septic shock and improve survival? | 20 | 477 | 490 | 967 | 4 |
| Nutrition/Rehabilitation | What is the prevalence, management and outcomes of post-intensive care syndrome? | 14 | 529 | 488 | 1017 | 6 |
|  | Can early mobilization preserve muscle mass, improve biomarkers and clinical outcomes in Asian ICUs? | 23 | 498 | 464 | 962 | 4 |
|  | What is the role of rehabilitation centers and post-ICU clinics in Asian ICUs? | 25 | 476 | 468 | 943 | 4 |
| Palliative Care | What is the current practice and challenges of do not resuscitate orders, limitation of therapy and end of life care in Asian ICUs? | 4 | 552 | 535 | 1088 | 8 |
| Respiratory Care/Failure | What is the incidence, management and outcomes of ARDS in Asian ICUs based on the new 2023 global definitions of ARDS? | 3 | 557 | 536 | 1092 | 6 |
| Shock/Intravenous Fluids | What are the best techniques and devices to assess fluid requirement in Asian ICUs? | 22 | 489 | 477 | 965 | 4 |
| Structure/Training/Staffing/Teamwork/Safety | What is the current and optimal staff to patient ratio and type of training across Asian ICUs from different income settings and types of ICU? | 6 | 523 | 534 | 1057 | 10 |
|  | How does organization factors such as referral system, open/closed, ICU/hospital bed capacity affect patient outcomes in Asian ICUs? | 12 | 517 | 516 | 1033 | 6 |
|  | What is the prevalence and factors associated with burn out amongst healthcare staff in Asian ICUs? | 8 | 514 | 528 | 1042 | 5 |
| Ultrasound | Can point of care ultrasound improve diagnosis and management of critically ill patients? | 19 | 514 | 479 | 992 | 5 |

The importance and feasibility score rating of 26 popular summarized research questions by National/Regional representatives. The overall priority score was calculated by the summation of the importance and feasibility score. Priority rank was ordered by the priority score of each summarized research question (highest priority is ranked 1). Popularity is calculated by counting the number of mentions for each summarized research question from all the 408 original research question suggestions. AKI, acute kidney injury; ARDS, acute respiratory distress syndrome; ECLS, extracorporeal life support; ICU, intensive care unit.

**Supplementary Table 6 Importance Criteria Component Scores**

| **Theme** | **Question** | Novelty | Uniqueness | Up-leveling | Inclusiveness | Impact | Anchor | Generalizability | Burden |
| --- | --- | --- | --- | --- | --- | --- | --- | --- | --- |
| AKI/Renal Replacement Therapy | What is the incidence, pathophysiology and outcomes of AKI in Asian ICUs? | 452 | 500 | 548 | 500 | 529 | 548 | 538 | 500 |
| Cardiology/Cardiothoracic/Cardiac Arrest | What is the current practice and cost-effectiveness of post-cardiac arrest care and advanced neuro-prognostication in Asian ICUs across all income settings? | 514 | 500 | 490 | 481 | 471 | 529 | 490 | 505 |
| Delirium/Sedation | What is the optimal use of sedation in the ICU? | 481 | 462 | 495 | 462 | 529 | 514 | 490 | 514 |
|  | What pharmacological and non-pharmacological strategies can prevent and treat delirium in ICU patients? | 486 | 481 | 514 | 448 | 486 | 514 | 524 | 510 |
|  | What is the incidence of delirium and current practice on delirium identification, prevention and treatment in Asian ICUs? | 486 | 486 | 552 | 467 | 505 | 495 | 500 | 505 |
| General ICU Care | Can artificial intelligence predict and improve outcomes of ICU patients across all income settings? | 543 | 481 | 476 | 462 | 562 | 452 | 457 | 519 |
|  | What is the prevalence and risk factors for venous thromboembolism in Asian ICUs? | 476 | 467 | 505 | 443 | 500 | 486 | 471 | 481 |
| Infection/Sepsis | What is the prevalence and outcomes of multidrug resistant infections and sepsis in Asian ICUs? | 567 | 576 | 610 | 543 | 610 | 552 | 581 | 581 |
|  | What is the optimal way to select, initiate, dose, administer and stop antibiotic therapy in Asian ICUs? | 524 | 524 | 529 | 514 | 562 | 533 | 548 | 562 |
|  | What the is prevalence, resistance pattern, management and outcomes of invasive fungal sepsis in Asian ICUs? | 533 | 552 | 562 | 519 | 567 | 500 | 552 | 543 |
|  | What is the incidence and mortality of sepsis and septic shock in Asia? | 510 | 495 | 567 | 552 | 552 | 529 | 548 | 557 |
|  | How is sepsis diagnosed in Asian ICUs and what are the factors associated with early detection? | 529 | 524 | 548 | 510 | 524 | 505 | 533 | 533 |
|  | Are anti-inflammatory drugs and immune modulator useful in sepsis? | 471 | 433 | 486 | 414 | 495 | 429 | 457 | 500 |
|  | What is the role of extracorporeal purification or plasma exchange in sepsis? | 505 | 443 | 510 | 471 | 524 | 462 | 457 | 510 |
|  | What is the current practice and challenges of antibiotic therapy in Asian ICUs? | 529 | 538 | 524 | 505 | 538 | 486 | 524 | 519 |
|  | What adjunctive treatments can shorten duration of septic shock and improve survival? | 495 | 438 | 495 | 452 | 510 | 462 | 476 | 486 |
| Nutrition/Rehabilitation | What is the prevalence, management and outcomes of post-intensive care syndrome? | 538 | 533 | 557 | 505 | 548 | 510 | 519 | 524 |
|  | Can early mobilization preserve muscle mass, improve biomarkers and clinical outcomes in Asian ICUs? | 510 | 467 | 543 | 476 | 529 | 462 | 486 | 514 |
|  | What is the role of rehabilitation centers and post-ICU clinics in Asian ICUs? | 476 | 481 | 500 | 452 | 514 | 471 | 457 | 452 |
| Palliative Care | What is the current practice and challenges of do not resuscitate orders, limitation of therapy and end of life care in Asian ICUs? | 567 | 576 | 567 | 543 | 576 | 529 | 519 | 543 |
| Respiratory Care/Failure | What is the incidence, management and outcomes of ARDS in Asian ICUs based on the new 2023 global definitions of ARDS? | 548 | 557 | 571 | 519 | 581 | 538 | 567 | 571 |
| Shock/Intravenous Fluids | What are the best techniques and devices to assess fluid requirement in Asian ICUs? | 486 | 448 | 510 | 486 | 505 | 500 | 486 | 490 |
| Structure/Training/Staffing/Teamwork/Safety | What is the current and optimal staff to patient ratio and type of training across Asian ICUs from different income settings and types of ICU? | 543 | 552 | 524 | 505 | 543 | 490 | 533 | 490 |
|  | How does organization factors such as referral system, open/closed, ICU/hospital bed capacity affect patient outcomes in Asian ICUs? | 529 | 538 | 519 | 490 | 543 | 490 | 519 | 510 |
|  | What is the prevalence and factors associated with burn out amongst healthcare staff in Asian ICUs? | 481 | 533 | 533 | 471 | 538 | 510 | 524 | 524 |
| Ultrasound | Can point of care ultrasound improve diagnosis and management of critically ill patients? | 524 | 505 | 533 | 514 | 543 | 476 | 481 | 533 |

The mean ratings of individual components of the importance criteria for each of the 26 popular summarized research questions by National/Regional representatives. AKI, acute kidney injury; ARDS, acute respiratory distress syndrome; ECLS, extracorporeal life support; ICU, intensive care unit.

**Supplementary Table 7 Feasibility Criteria Component Scores**

| **Theme** | **Question** | Cultural | Expertise | Time Frame | Regional Differences | Recruitment | Ethics | Cost | Resources |
| --- | --- | --- | --- | --- | --- | --- | --- | --- | --- |
| AKI/Renal Replacement Therapy | What is the incidence, pathophysiology and outcomes of AKI in Asian ICUs? | 505 | 543 | 543 | 533 | 500 | 529 | 481 | 510 |
| Cardiology/Cardiothoracic/Cardiac Arrest | What is the current practice and cost-effectiveness of post-cardiac arrest care and advanced neuro-prognostication in Asian ICUs across all income settings? | 495 | 481 | 529 | 495 | 510 | 490 | 471 | 524 |
| Delirium/Sedation | What is the optimal use of sedation in the ICU? | 486 | 510 | 514 | 519 | 486 | 519 | 486 | 552 |
|  | What pharmacological and non-pharmacological strategies can prevent and treat delirium in ICU patients? | 505 | 476 | 529 | 495 | 495 | 505 | 486 | 524 |
|  | What is the incidence of delirium and current practice on delirium identification, prevention and treatment in Asian ICUs? | 557 | 538 | 543 | 529 | 519 | 529 | 481 | 533 |
| General ICU Care | Can artificial intelligence predict and improve outcomes of ICU patients across all income settings? | 395 | 386 | 514 | 448 | 457 | 471 | 433 | 462 |
|  | What is the prevalence and risk factors for venous thromboembolism in Asian ICUs? | 524 | 495 | 519 | 495 | 514 | 514 | 448 | 486 |
| Infection/Sepsis | What is the prevalence and outcomes of multidrug resistant infections and sepsis in Asian ICUs? | 529 | 548 | 571 | 548 | 538 | 548 | 510 | 581 |
|  | What is the optimal way to select, initiate, dose, administer and stop antibiotic therapy in Asian ICUs? | 543 | 538 | 552 | 548 | 524 | 548 | 495 | 571 |
|  | What the is prevalence, resistance pattern, management and outcomes of invasive fungal sepsis in Asian ICUs? | 500 | 500 | 548 | 519 | 524 | 519 | 486 | 533 |
|  | What is the incidence and mortality of sepsis and septic shock in Asia? | 576 | 562 | 567 | 567 | 552 | 562 | 505 | 581 |
|  | How is sepsis diagnosed in Asian ICUs and what are the factors associated with early detection? | 514 | 510 | 529 | 529 | 533 | 524 | 481 | 548 |
|  | Are anti-inflammatory drugs and immune modulator useful in sepsis? | 471 | 457 | 519 | 476 | 481 | 510 | 467 | 538 |
|  | What is the role of extracorporeal purification or plasma exchange in sepsis? | 438 | 429 | 490 | 429 | 457 | 462 | 438 | 500 |
|  | What is the current practice and challenges of antibiotic therapy in Asian ICUs? | 548 | 557 | 543 | 533 | 519 | 529 | 500 | 557 |
|  | What adjunctive treatments can shorten duration of septic shock and improve survival? | 448 | 452 | 514 | 490 | 500 | 514 | 471 | 529 |
| Nutrition/Rehabilitation | What is the prevalence, management and outcomes of post-intensive care syndrome? | 467 | 457 | 529 | 471 | 495 | 524 | 457 | 500 |
|  | Can early mobilization preserve muscle mass, improve biomarkers and clinical outcomes in Asian ICUs? | 443 | 429 | 471 | 457 | 476 | 500 | 457 | 476 |
|  | What is the role of rehabilitation centers and post-ICU clinics in Asian ICUs? | 438 | 438 | 510 | 448 | 462 | 490 | 462 | 495 |
| Palliative Care | What is the current practice and challenges of do not resuscitate orders, limitation of therapy and end of life care in Asian ICUs? | 538 | 562 | 548 | 519 | 514 | 533 | 510 | 557 |
| Respiratory Care/Failure | What is the incidence, management and outcomes of ARDS in Asian ICUs based on the new 2023 global definitions of ARDS? | 538 | 557 | 571 | 529 | 514 | 524 | 486 | 567 |
| Shock/Intravenous Fluids | What are the best techniques and devices to assess fluid requirement in Asian ICUs? | 462 | 438 | 519 | 467 | 490 | 486 | 443 | 510 |
| Structure/Training/Staffing/Teamwork/Safety | What is the current and optimal staff to patient ratio and type of training across Asian ICUs from different income settings and types of ICU? | 529 | 543 | 533 | 548 | 514 | 557 | 519 | 529 |
|  | How does organization factors such as referral system, open/closed, ICU/hospital bed capacity affect patient outcomes in Asian ICUs? | 519 | 538 | 524 | 524 | 510 | 510 | 481 | 524 |
|  | What is the prevalence and factors associated with burn out amongst healthcare staff in Asian ICUs? | 548 | 552 | 514 | 538 | 543 | 514 | 500 | 514 |
| Ultrasound | Can point of care ultrasound improve diagnosis and management of critically ill patients? | 481 | 457 | 524 | 481 | 419 | 490 | 467 | 510 |

The mean ratings of individual components of the feasibility criteria for each of the 26 popular summarized research questions by National/Regional representatives. AKI, acute kidney injury; ARDS, acute respiratory distress syndrome; ECLS, extracorporeal life support; ICU, intensive care unit.

**Supplementary Figure 1 Distribution of Importance and Feasibility Likert Responses**

Question 1: What is the incidence, pathophysiology and outcomes of AKI in Asian ICUs?

**
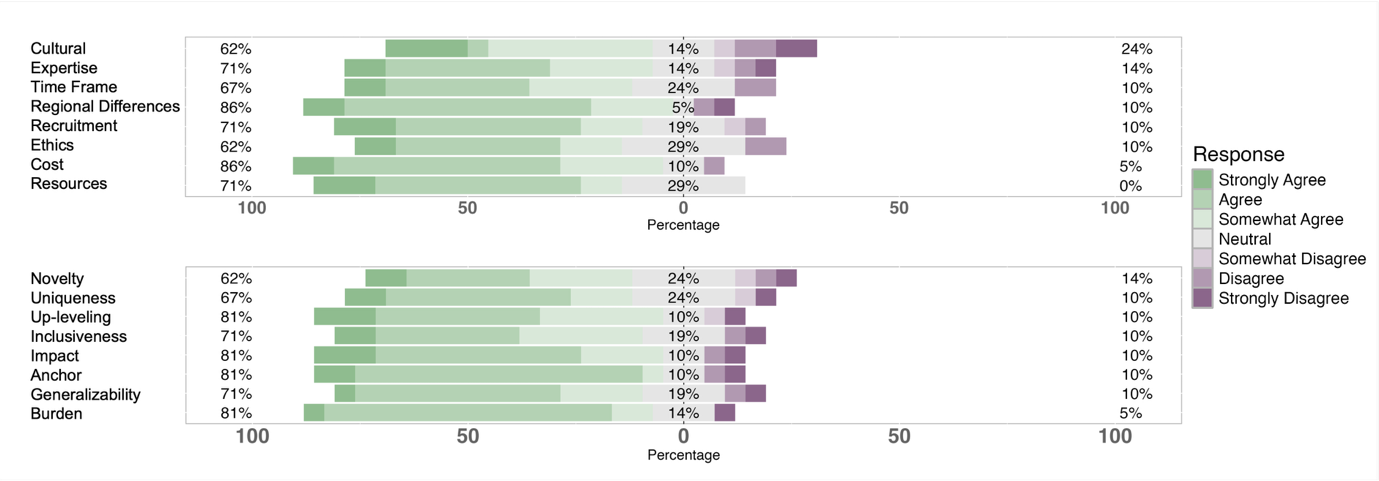
**

Question 2: What is the current practice and cost-effectiveness of post-cardiac arrest care and advanced neuro-prognostication in Asian ICUs across all income settings?

**
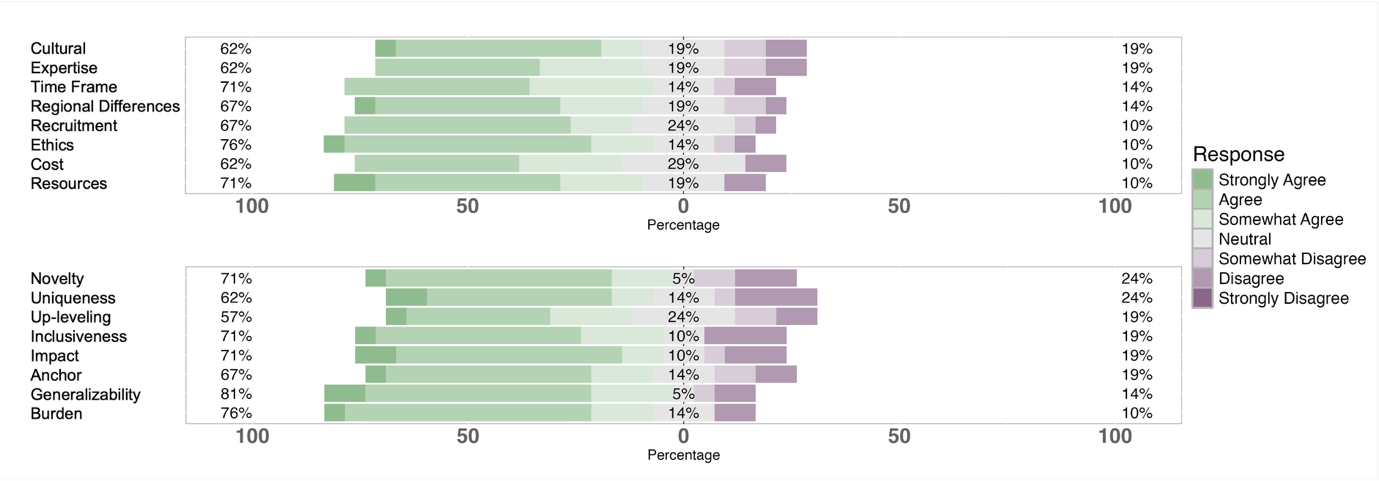
**

Question 3: What is the optimal use of sedation in the ICU?

**
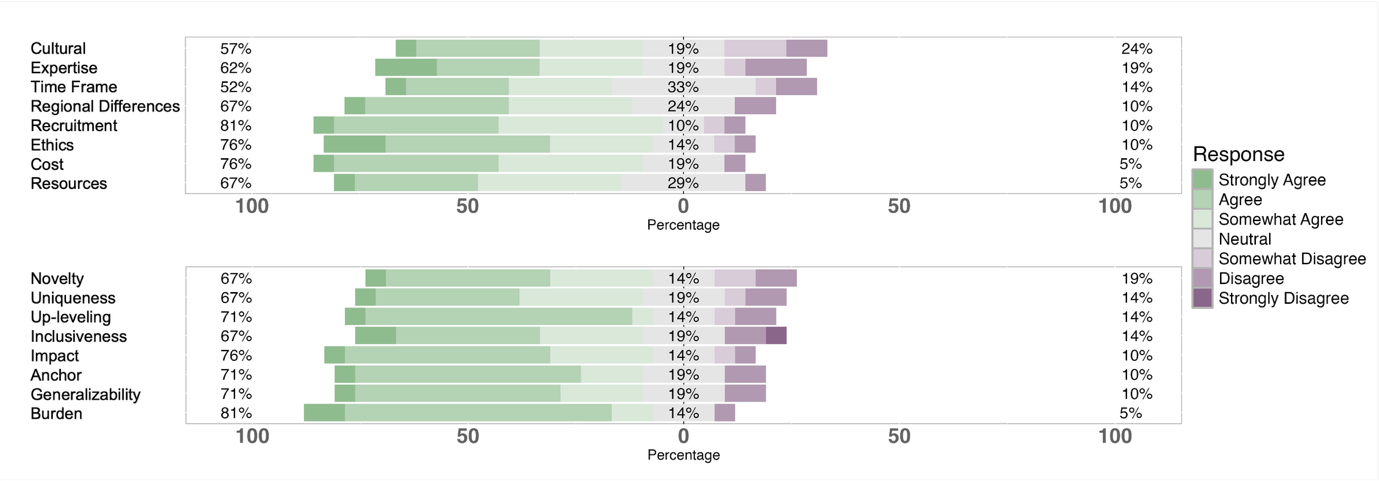
**

Question 4: What pharmacological and non-pharmacological strategies can prevent and treat delirium in ICU patients?


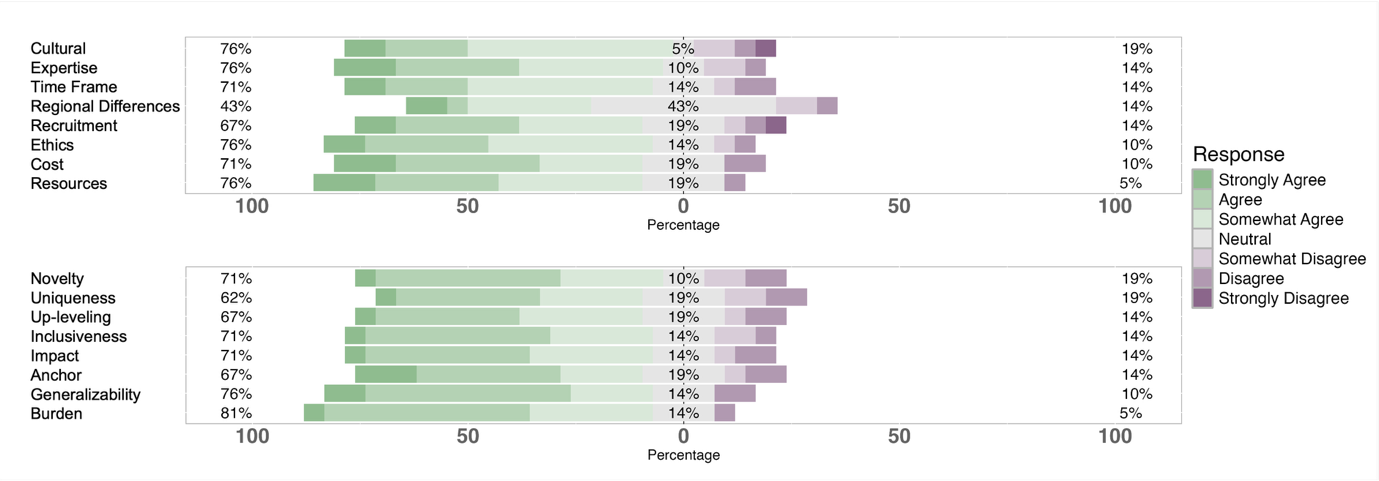


Question 5: What is the incidence of delirium and current practice on delirium identification, prevention and treatment in Asian ICUs?


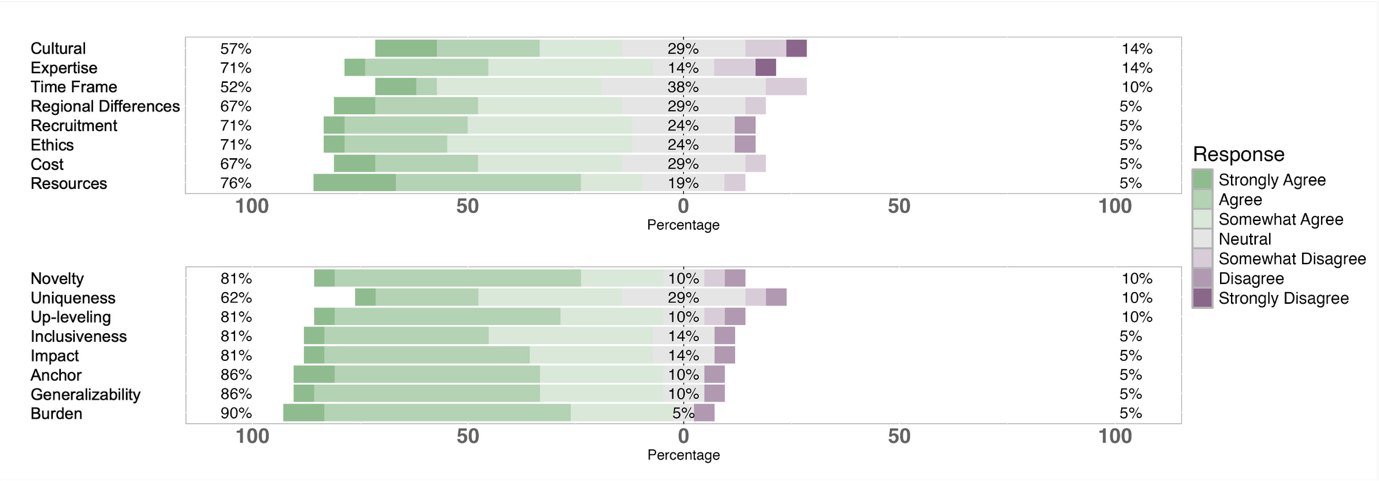


Question 6: Can artificial intelligence predict and improve outcomes of ICU patients across all income settings?


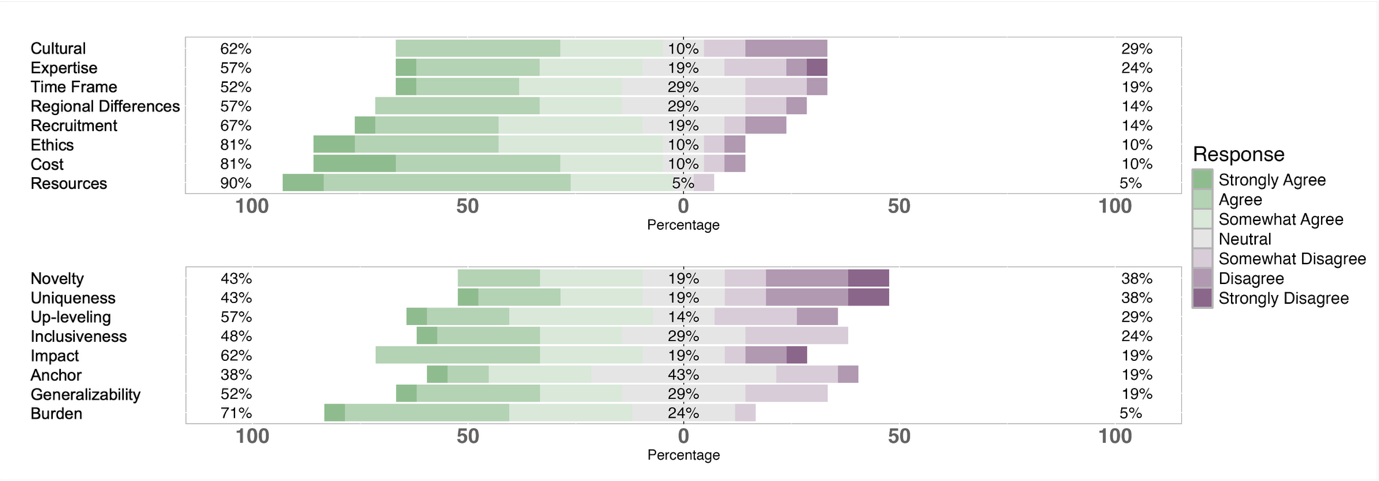


Question 7: What is the prevalence and risk factors for venous thromboembolism in Asian ICUs?
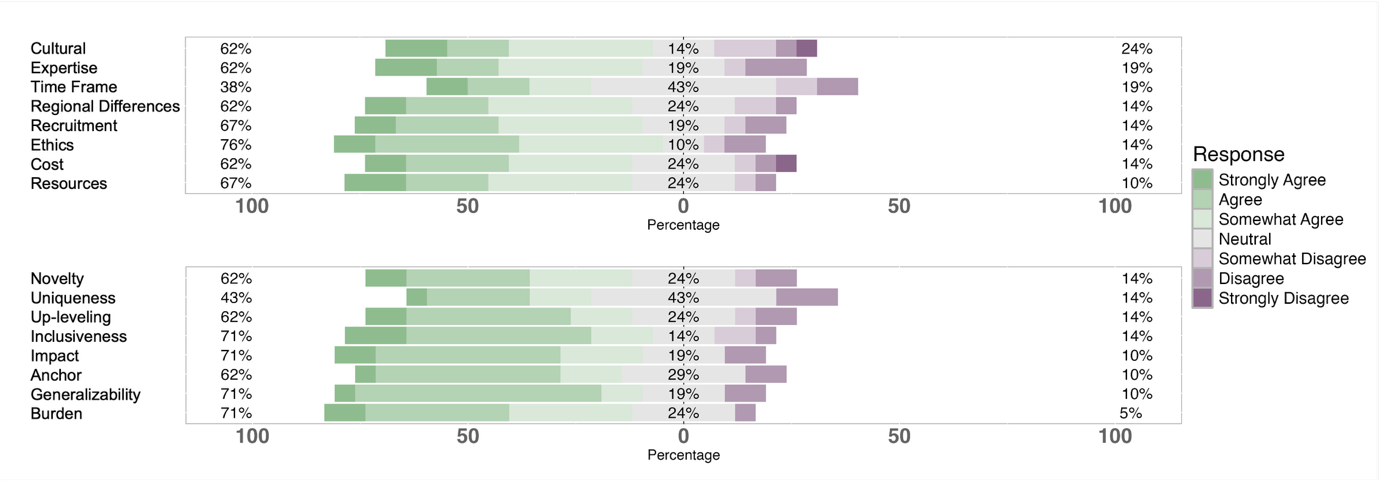


Question 8: What is the prevalence and outcomes of multidrug resistant infections and sepsis in Asian ICUs?


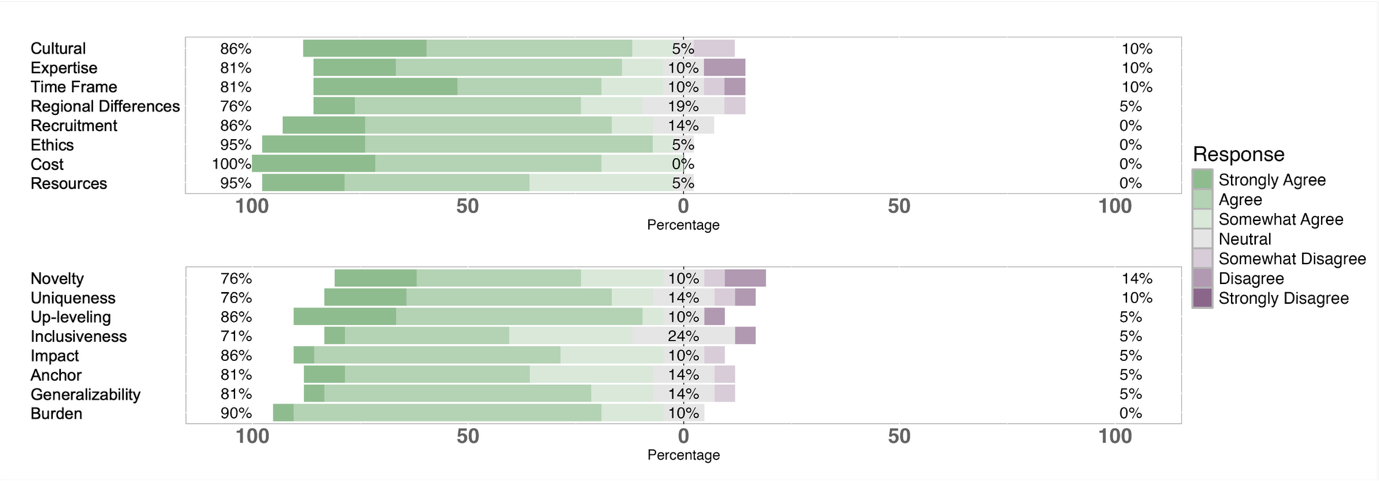


Question 9: What is the optimal way to select, initiate, dose, administer and stop antibiotic therapy in Asian ICUs?


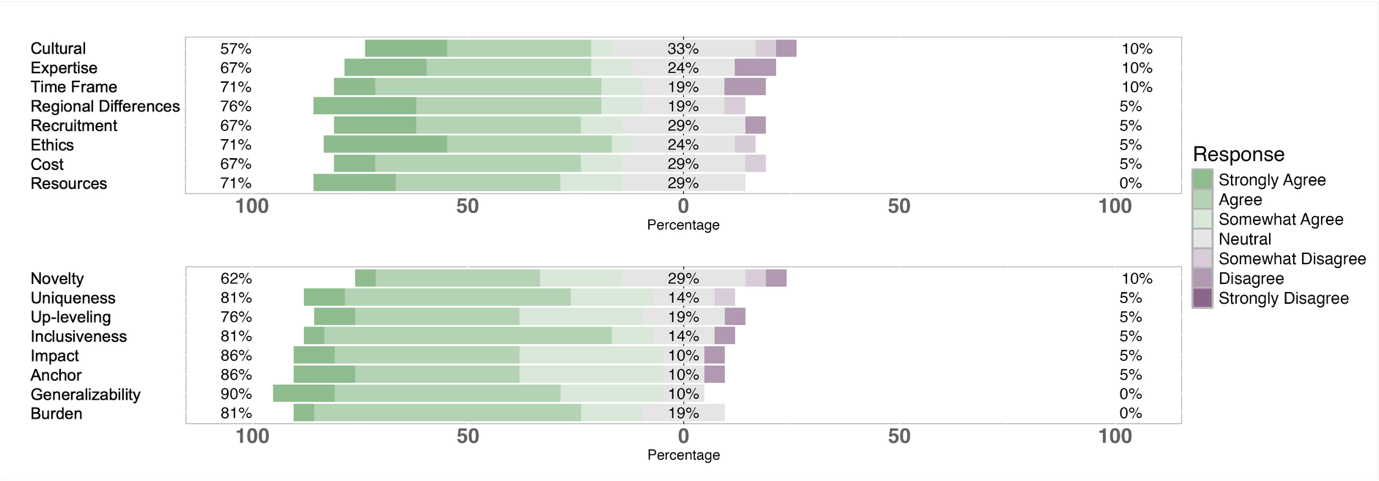


Question 10: What the is prevalence, resistance pattern, management and outcomes of invasive fungal sepsis in Asian ICUs?


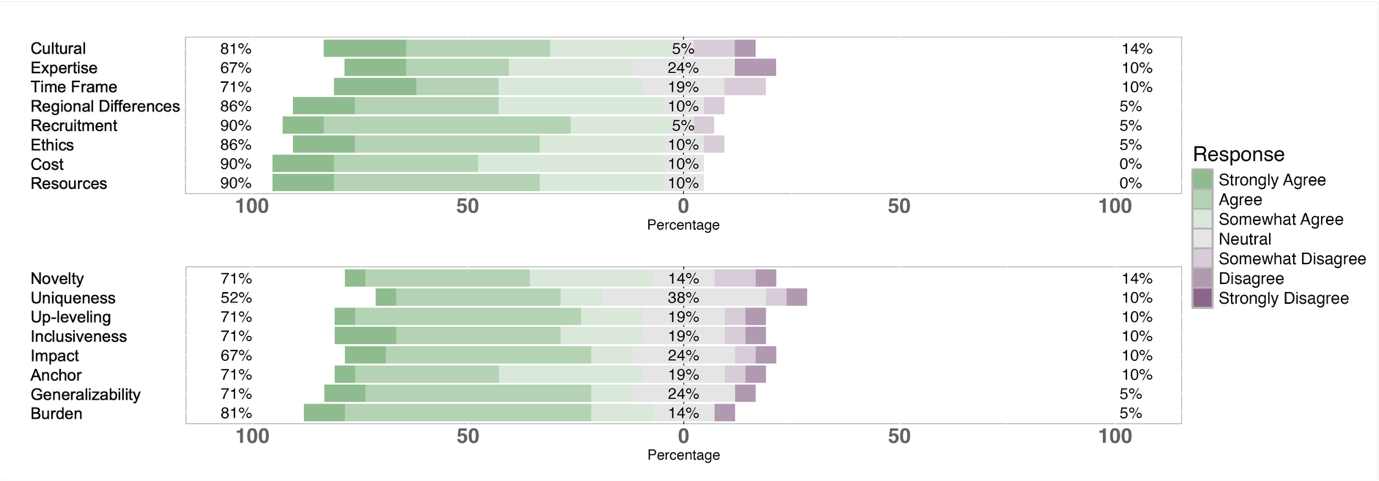


Question 11: What is the incidence and mortality of sepsis and septic shock in Asia?


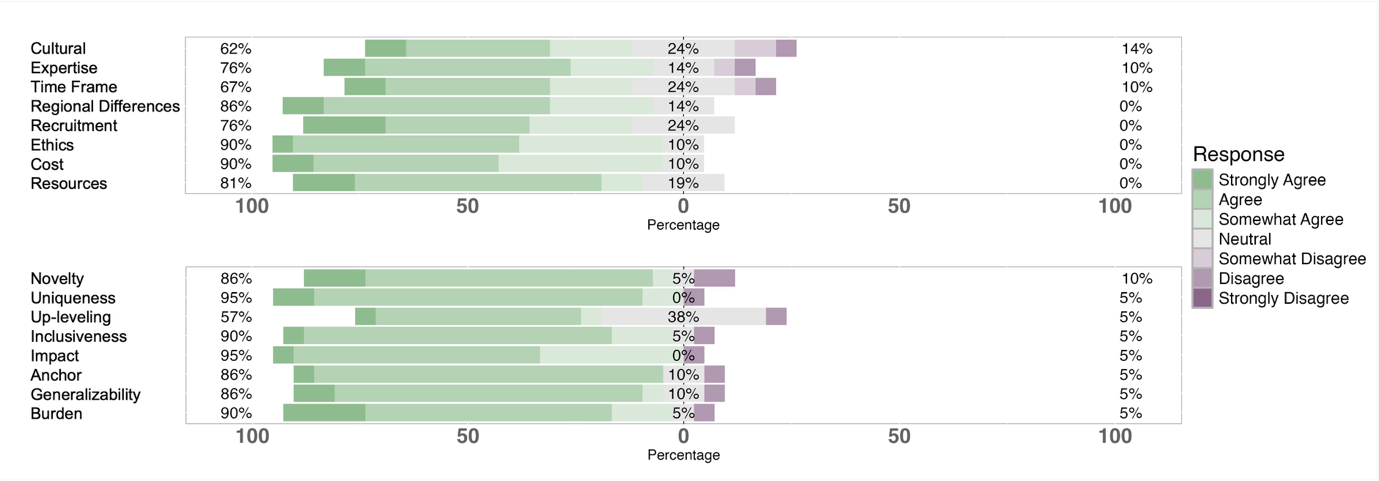


Question 12: How is sepsis diagnosed in Asian ICUs and what are the factors associated with early detection?


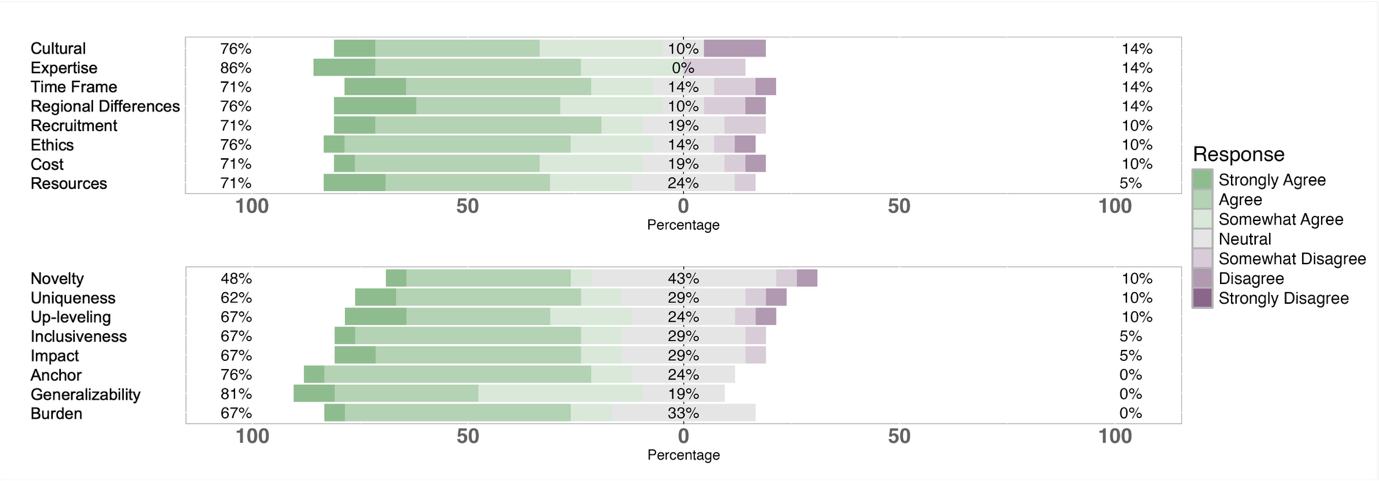


Question 13: Are anti-inflammatory drugs and immune modulator useful in sepsis?


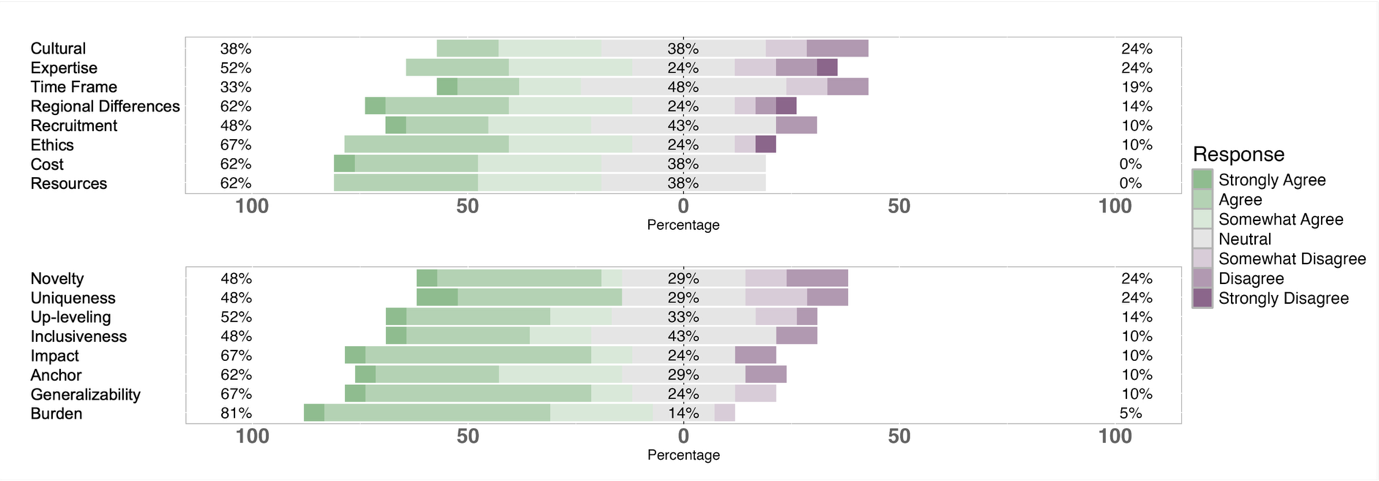


Question 14: What is the role of extracorporeal purification or plasma exchange in sepsis?


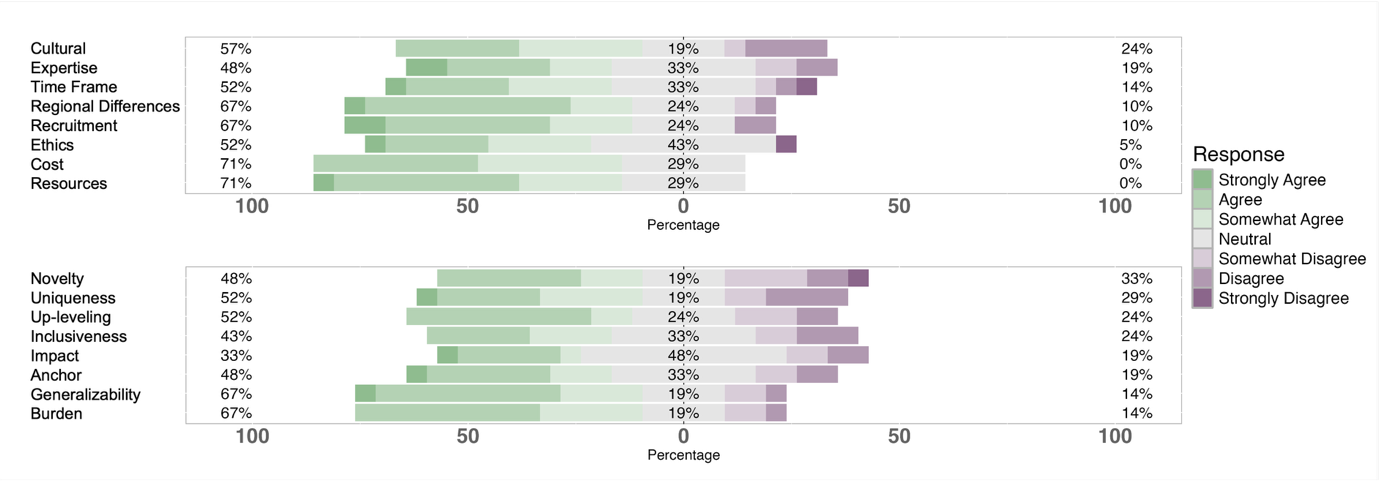


Question 15: What is the current practice and challenges of antibiotic therapy in Asian ICUs?


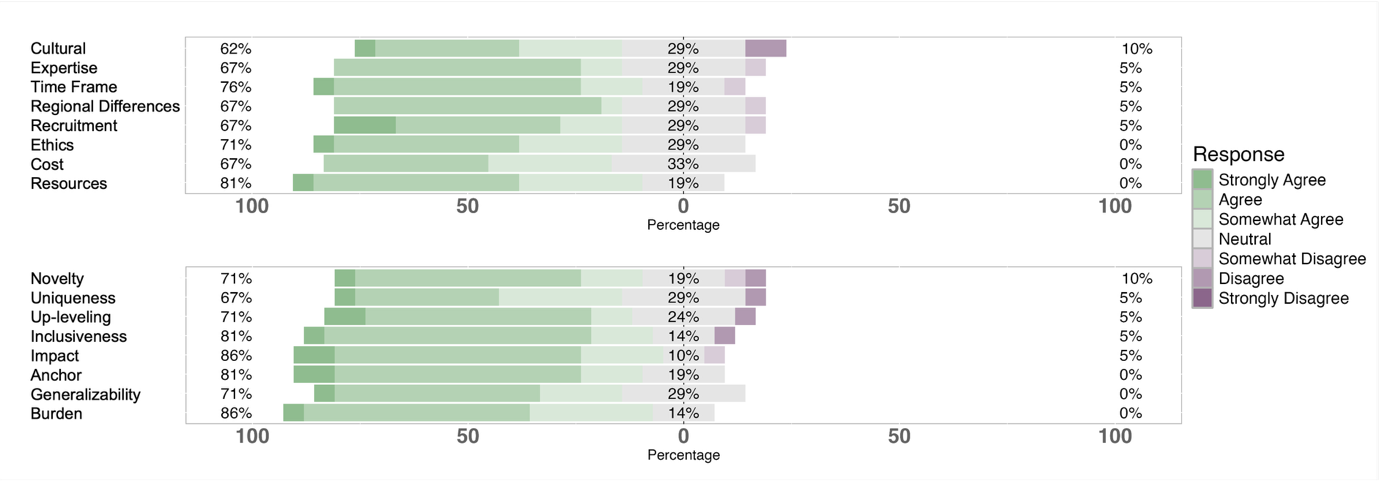


Question 16: What adjunctive treatments can shorten duration of septic shock and improve survival?


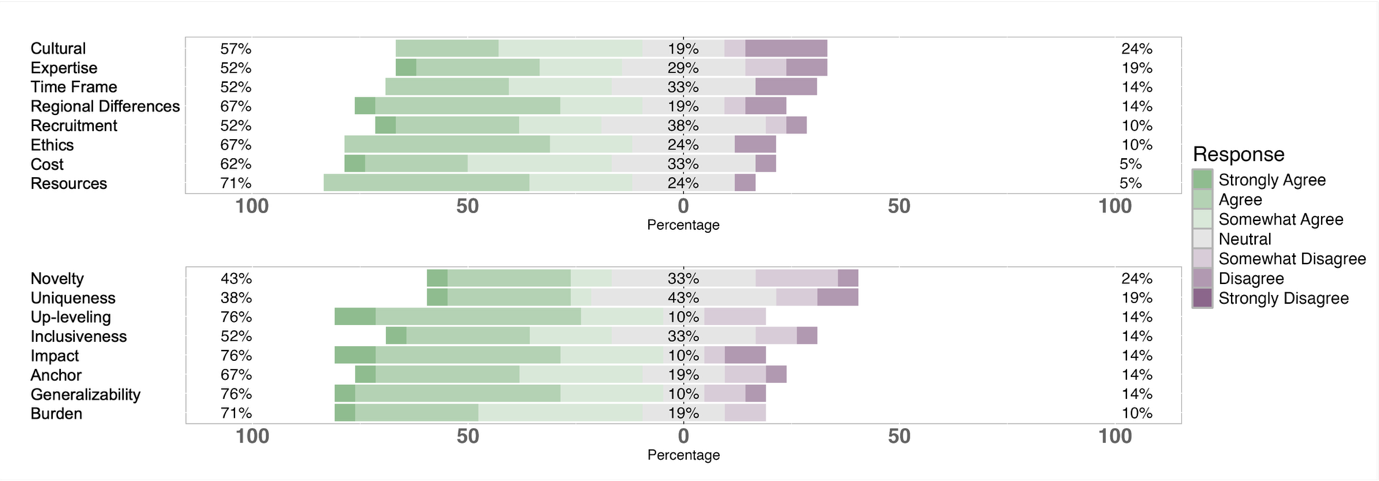


Question 17: What is the prevalence, management and outcomes of post-intensive care syndrome?


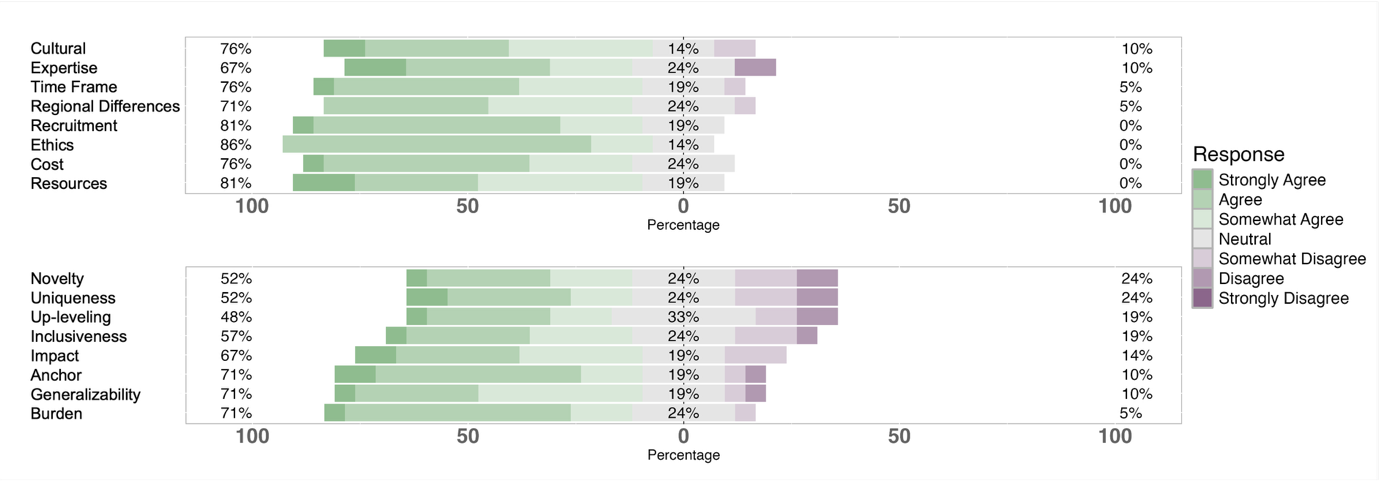


Question 18: Can early mobilization preserve muscle mass, improve biomarkers and clinical outcomes in Asian ICUs?


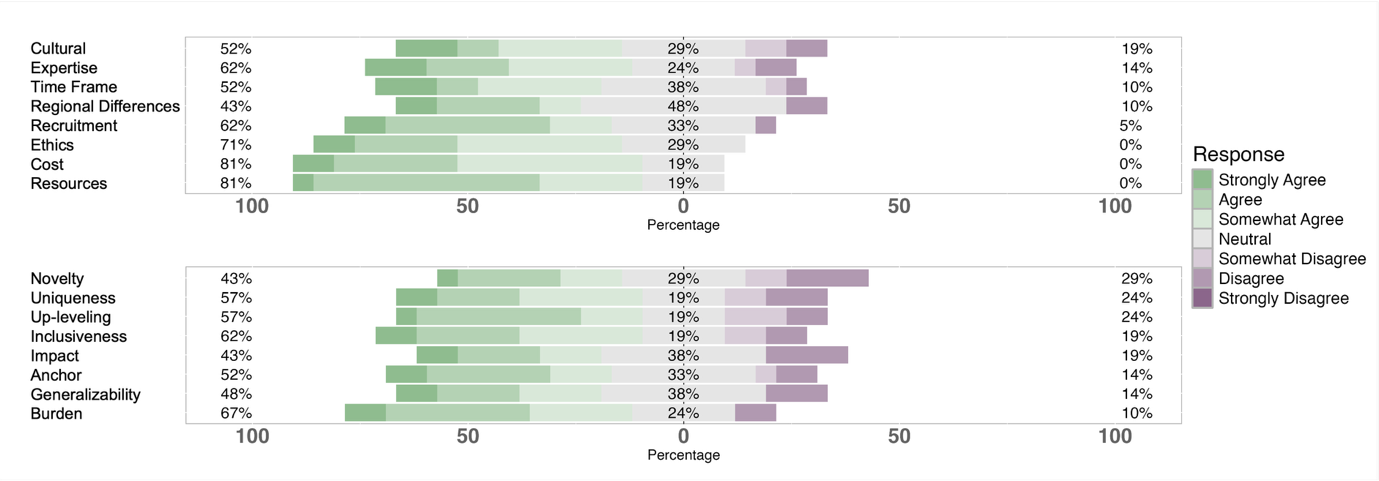


Question 19: What is the role of rehabilitation centers and post-ICU clinics in Asian ICUs?


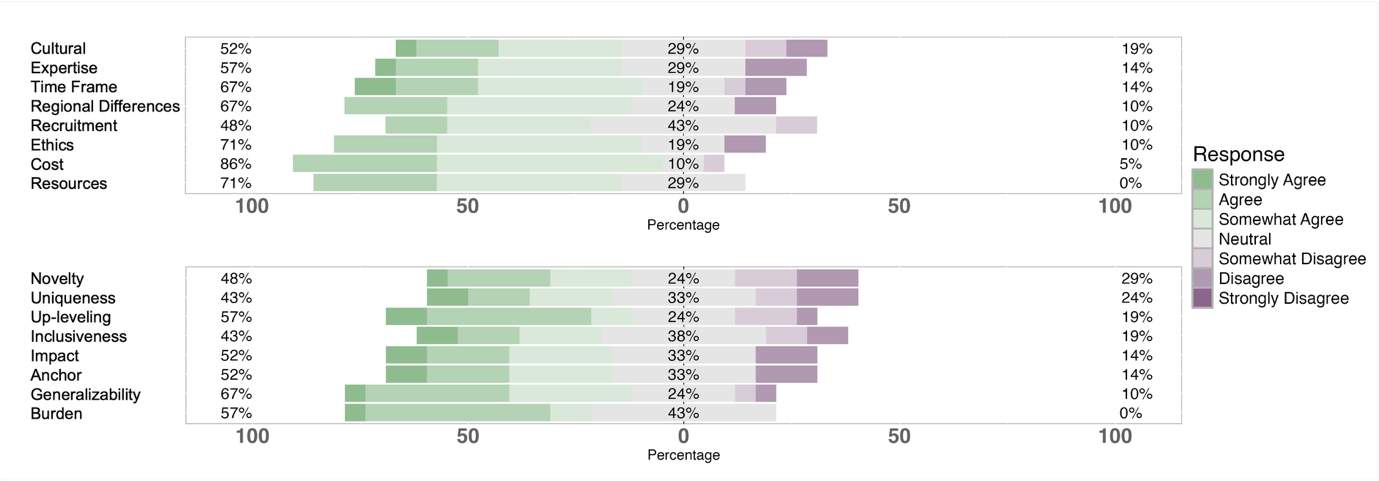


Question 20: What is the current practice and challenges of do not resuscitate orders, limitation of therapy and end of life care in Asian ICUs?


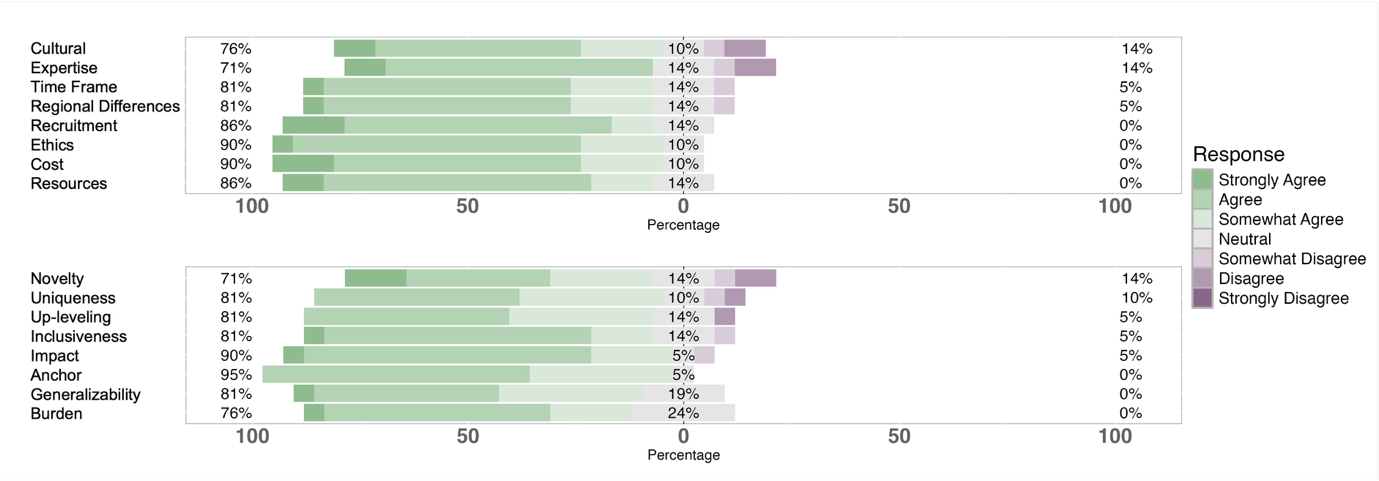


Question 21: What is the incidence, management and outcomes of ARDS in Asian ICUs based on the new 2023 global definitions of ARDS?


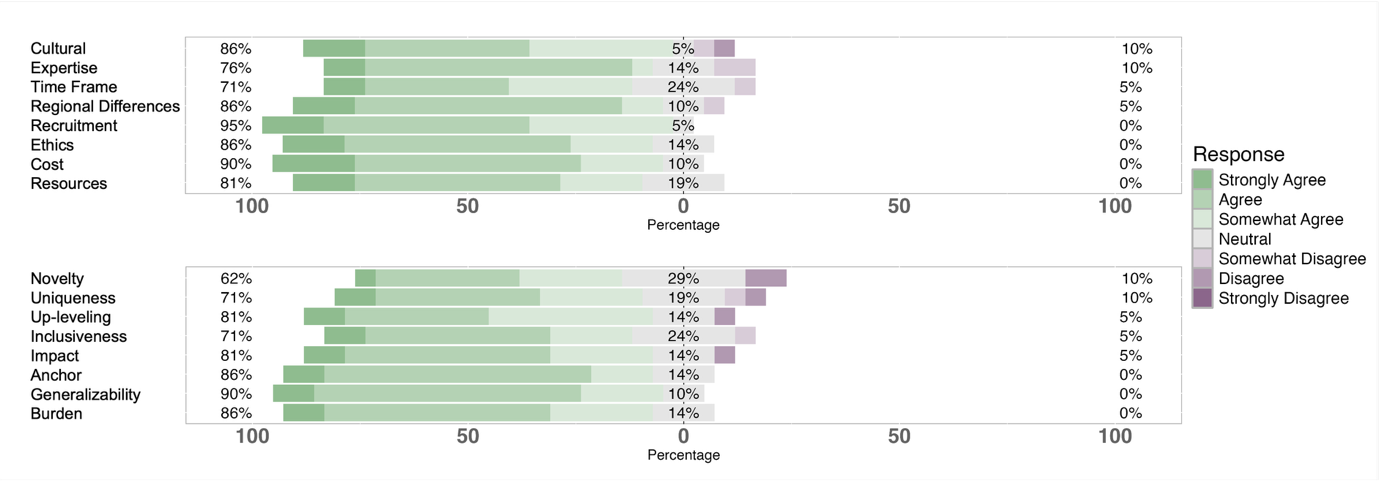


Question 22: What are the best techniques and devices to assess fluid requirement in Asian ICUs?


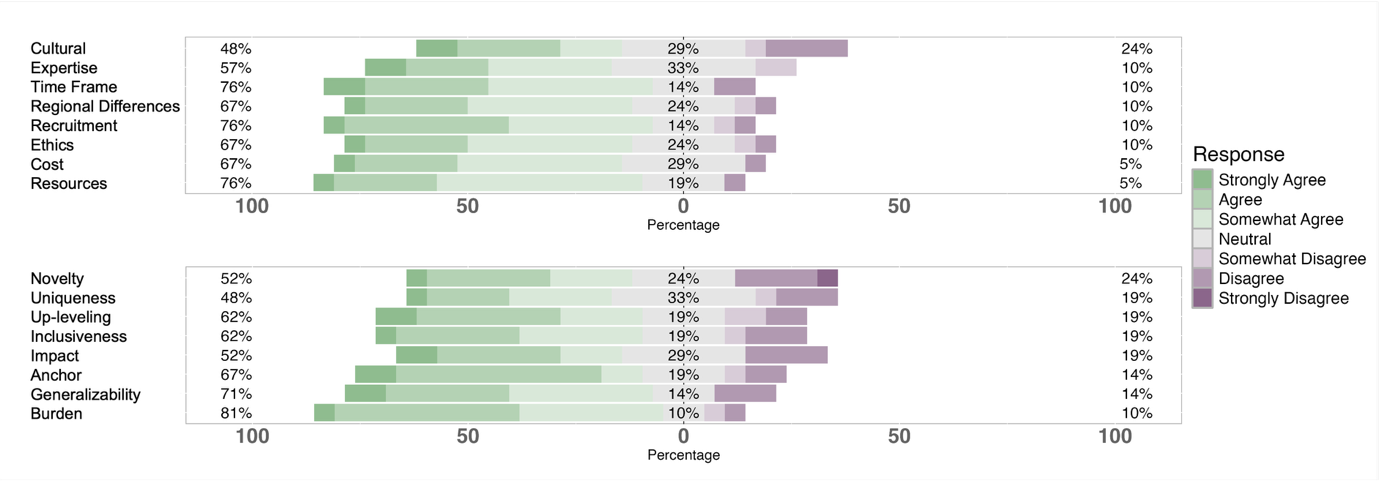


Question 23: What is the current and optimal staff to patient ratio and type of training across Asian ICUs from different income settings and types of ICU?


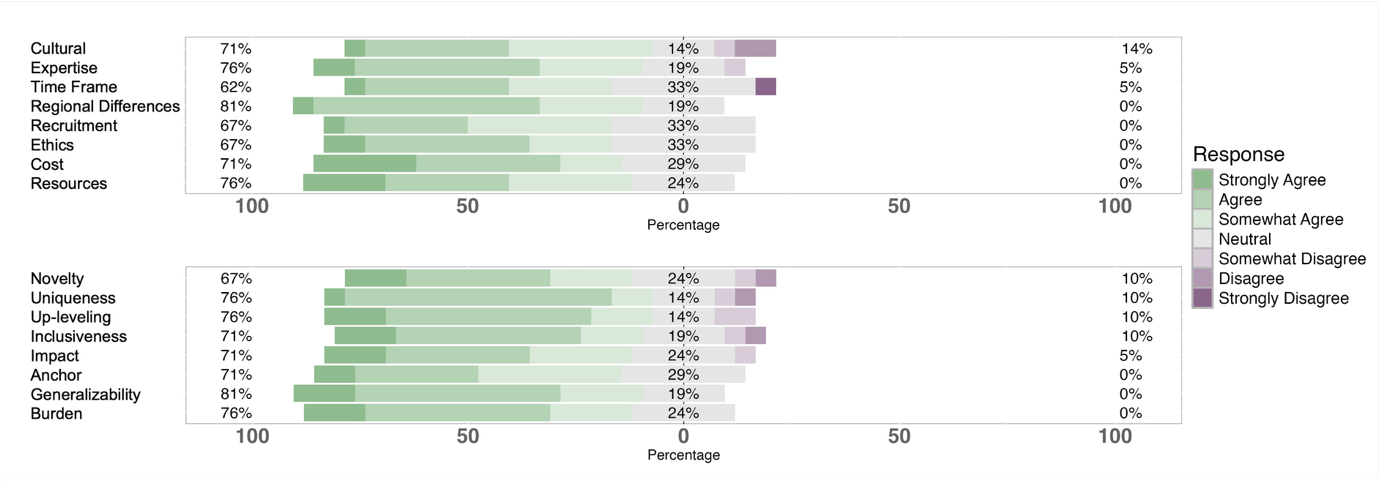


Question 24: How does organization factors such as referral system, open/closed, ICU/hospital bed capacity affect patient outcomes in Asian ICUs?


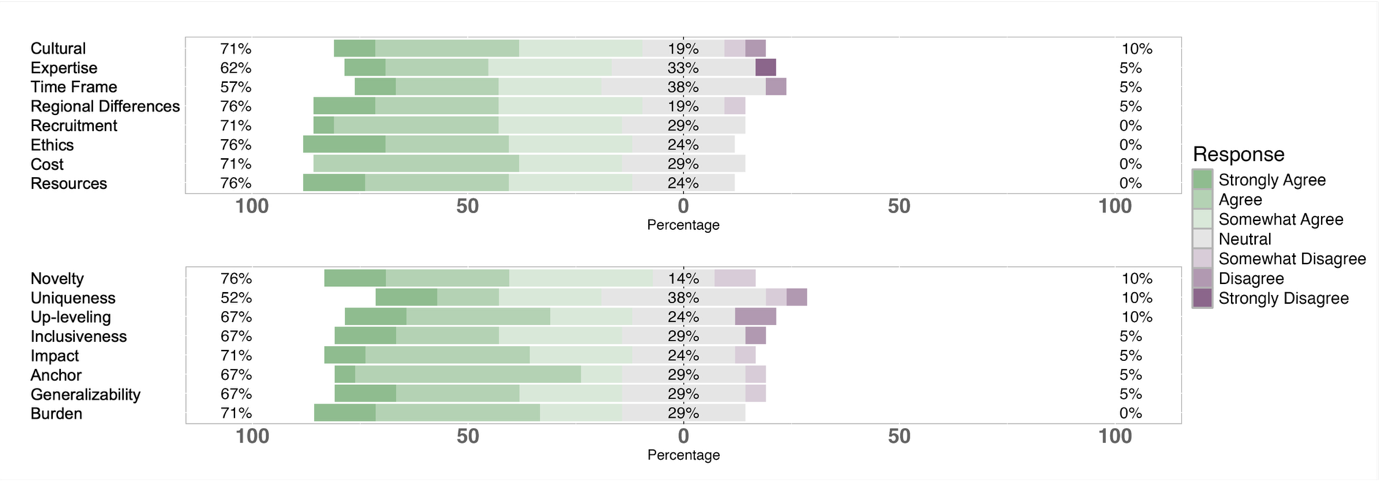


Question 25: What is the prevalence and factors associated with burn out amongst healthcare staff in Asian ICUs?


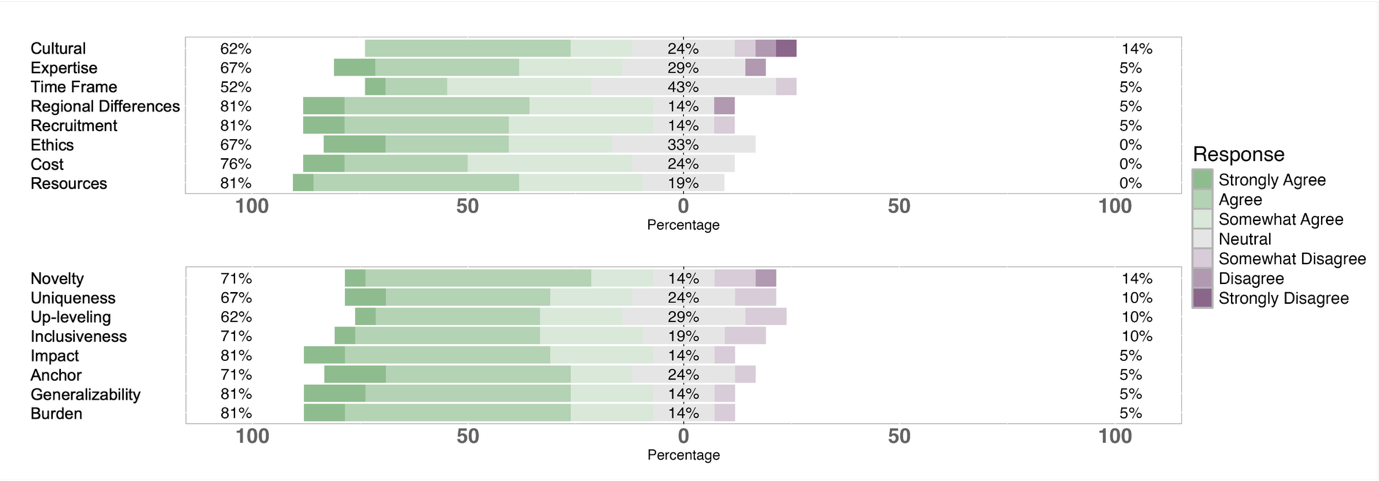


Question 26: Can point of care ultrasound improve diagnosis and management of critically ill patients?

**
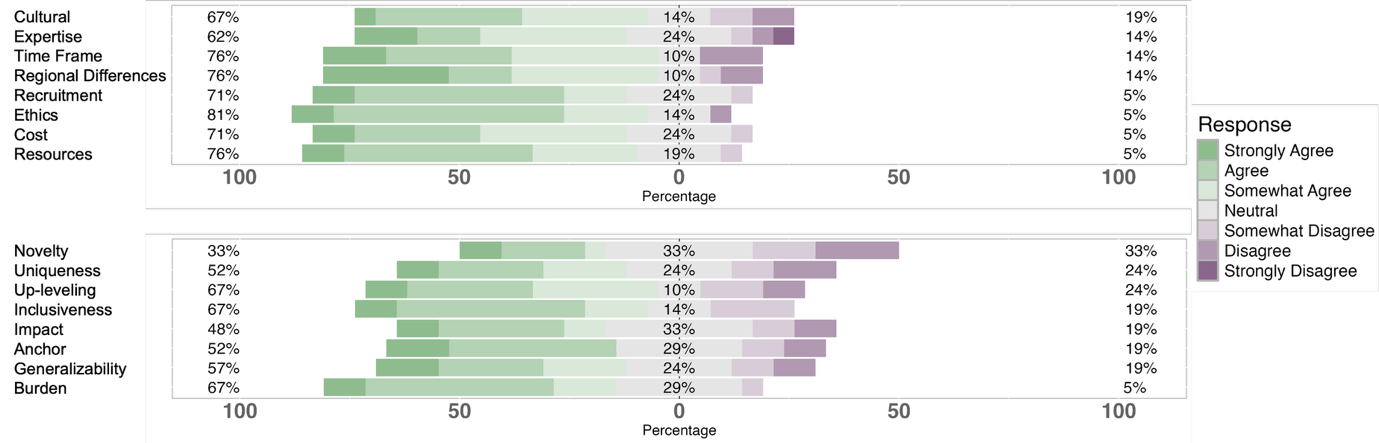
**

The distribution of Likert ratings for all components of importance and feasibility criteria for 26 most popular summary research questions.
